# Multiscale Brownian dynamics simulations reveal effects of macromolecular crowding on protein–ligand association

**DOI:** 10.1073/pnas.2617950123

**Published:** 2026-10-30

**Authors:** Riccardo Beccaria, Abraham Muñiz-Chicharro, Rebecca C. Wade

**Affiliations:** ^a^ Molecular and Cellular Modeling Group, Heidelberg Institute for Theoretical Studies, Heidelberg 69118, Germany; ^b^Faculty of Physics, Heidelberg University, Heidelberg 69120, Germany; ^c^Faculties of Engineering Sciences and Biosciences, Center for Molecular Biology of Heidelberg University (ZMBH), DKFZ-ZMBH Alliance, Interdisciplinary Center for Scientific Computing, Heidelberg University, Heidelberg 69120, Germany

**Keywords:** association rate constant, multiresolution Brownian dynamics simulation, macromolecular crowding, protein–ligand complex

## Abstract

Macromolecular crowding is known to influence the diffusive, binding, kinetic, and equilibrium properties of proteins in a range of environments such as occur inside biological cells or in highly concentrated solutions of protein therapeutics. However a quantitative understanding of the effects of macromolecular crowding on the rates of protein-small molecule binding is lacking. Here, we present an adaptive model to simulate the association of two solutes in a macromolecularly crowded environment using Brownian dynamics and to compute the corresponding bimolecular association rate constant. The adaptive model represents interactions with atomistic detail for short-range crowder–noncrowder interactions, while using a coarse-grained approach for long-range crowder–noncrowder interactions and all crowder–crowder interactions. This multiscale approach is integrated in the Simulation of Diffusional Association (SDA) software for Brownian dynamics simulation of biomolecular diffusion. We applied this model to compute the rates of association for two contrasting protein-small molecule inhibitor complexes in environments varying in macromolecular crowder type and density, and we used Markov State modeling of the encounter trajectories to analyze the binding pathways. The results reveal competing effects: While the presence of the crowders can hinder ligand binding by reducing the translational diffusion rates of the solutes and obstructing the protein binding sites, crowders can also enhance the bimolecular association rate by other mechanisms, including channeling and the caging effect.

## Significance

Macromolecular crowding, such as the dense packing of biomolecules in cellular environments, can alter how proteins and small molecules interact, yet its effects on binding rates and pathways remain poorly understood. We developed a multiresolution Brownian dynamics framework that efficiently combines atomistic and coarse-grained representations to simulate molecular association in crowded environments. The method enables prediction of binding rates and analysis of binding mechanisms under crowding conditions. Simulations of two representative protein-small molecule systems reveal that macromolecular crowding can both suppress and enhance association rates through competing mechanisms, including steric obstruction, molecular channeling, and caging effects. Moreover, macromolecular crowding alters binding pathways. These results highlight the importance of considering the impact of crowded environments on biomolecular and therapeutic interactions.

The intracellular environment is highly crowded, with up to 40% of its volume occupied by macromolecules, such as proteins, nucleic acids, and carbohydrates (1, 2). Crowding impacts the behavior of proteins inside cells, influencing their motion, and their kinetic and equilibrium properties (3). Different studies have shown that an environment that is crowded with macromolecules can hinder protein diffusion (4–10), alter the phase separation of small proteins (11, 12), modulate enzyme activity (13–16), influence the self-assembly of proteins and DNA (17–22), affect intracellular transport (23), and alter protein folding stability (24, 25) and association rates (26–32).

The effects of crowding have been largely attributed to excluded volume due to steric exclusion by the crowder molecules, which affects the solvent volume available to the diffusing solutes. It can directly affect protein mobility with knock-on effects on cellular processes (33–36). However, numerous studies suggest that excluded volume alone cannot fully explain protein motion in crowded environments, and that, in addition to steric hindrance, specific interactions (such as electrostatic or hydrophobic forces) between proteins and crowders appear to play a critical role (37–41). Therefore, systematic methods are needed to study how these interactions, along with variations in crowding molecule size and heterogeneity, influence protein diffusion, and dynamics (42).

Molecular simulation techniques offer powerful tools to investigate these phenomena, enabling the detailed analysis of molecular trajectories and specific interactions in crowded environments (43–46). However, accurately simulating cellular crowding presents challenges (47–49): Due to the large number of molecules involved, the application of classical Molecular Dynamics (MD) simulations is highly demanding (50–52). Brownian Dynamics (BD) simulation is a valuable alternative approach that combines computational efficiency with the ability to capture key features of the molecules at atomistic resolution (53–57).

In BD simulations, the solvent is treated implicitly and the solutes follow a Brownian motion in which the displacement is given by both a drift term, which accounts for solute–solute and solute–solvent interactions, and a stochastic term that mimics the collision between solutes and solvent molecules (53, 58). In BD simulations, the solutes are often treated as rigid bodies, which together with the implicit solvent representation, reduces computational time by reducing the number of degrees of freedom, thereby enabling simulations of larger systems over longer timescales (57).

Here, we developed an adaptive model to simulate macromolecular association processes in a crowded environment in order to compute the bimolecular association rate constant. In the adaptive model, molecular interactions are represented with atomistic resolution (near-field force field) when crowders are near to the two associating molecules and with a coarser grained force field (far-field force field) when they are farther away. The transition from one resolution to the other is smooth, ensuring continuity in the potentials and forces. We integrated this adaptive model into the latest release of the Simulation of Diffusional Association software (SDA 7) (53). We employed this dual-resolution model to investigate the binding of two contrasting protein-small molecule ligand complexes under different crowding conditions. These complexes were of trypsin with benzamidine, and of haspin kinase with 5-chlorotubercidin. These complexes were chosen to represent important features for protein–drug binding, namely, electrostatically steered and hydrophobically driven binding, respectively. Trypsin, a digestive protein, is a monomeric serine protease. Catalysis is supported by the electrostatic interactions between its acidic pocket and positively charged residues, lysine and arginine, of the target proteins. Benzamidine is a low molecular weight basic compound that competitively inhibits trypsin by binding in its acidic pocket (59). Haspin, a serine/threonine-protein kinase critical for mitosis, has emerged as a promising target in developing anticancer therapeutics due to its role in cell cycle regulation. 5-chlorotubercidin, an adenosine-analog, inhibits the enzyme by binding in its ATP-binding site (60). The association of each of these two complexes was simulated in the presence of a range of concentrations of model crowders that were either C60 fullerenes or (charged or neutralized) hen egg white lysozyme (HEWL) protein molecules or a mixture of the two. HEWL is an experimentally well-characterized protein for which a rigid body model is appropriate due to its high conformational stability (61). It was chosen to model the physical effects of dense cellular environments like biomolecular condensates. Because these biological compartments are frequently enriched with highly charged species (such as RNA-binding proteins), we systematically compared charged and neutralized HEWL models (62). This allowed us to identify how the electrostatic properties of crowders—not just their size and shape—modulate small-molecule diffusion and protein binding kinetics. C60 fullerene was selected to establish a direct methodological bridge with classical, idealized “hard-sphere” or “void” crowding models used in previous studies, e.g., refs. 16, 63, and 64. Furthermore, the choice of C60 is justified by its emerging prominence in bionanotechnology, where C60–protein combinations, including mixtures of C60 and HEWL, have been investigated for applications in nanomedicine and photodynamic therapy (65, 66). The BD simulations reveal how different effects of macromolecular crowding contribute to differing dependence of the bimolecular association rate constants on crowder concentration in the different systems.

## Theory

### Brownian Dynamics Simulation of the Diffusion of Solutes in the Presence of Crowder Molecules.

Brownian dynamics is a simulation method in which the motion of solute molecules is simulated using an implicit solvent representation, where solutes are often represented as rigid bodies (57). By implicitly representing water molecules and ions, and neglecting internal degrees of freedom, a longer time step can be used in simulations, and interactions can be calculated from precomputed grids. This allows systems to be studied on a longer time scale than is usually achievable with classical all-atom molecular dynamics simulations.

SDA 7 is a BD simulation software package (53) capable of handling many solutes by treating them as rigid bodies with atomistic details (67, 68). This software uses a simplified version of the algorithm proposed by Ermak and McCammon (69) to model the propagation of Brownian particles while including hydrodynamic interactions. The latter are calculated via a mean-field approximation, framework that has been extensively validated for crowded macromolecular environments (70). This mean field model defines the local occupied volume fraction $V_{i}^{\mathrm{frac}}$ of the solute *i* in the presence of other solutes *j* as

[1] $$\begin{array}{c} V_{i}^{\mathrm{frac}}=\frac{v_{i}+\sum_{j}v_{j}}{V_{i}}, \end{array}$$

where $v_{i}$ and $v_{j}$ are the volumes occupied by solutes *i* and *j*, respectively, and $V_{i}$ is the total spherical volume defined by four times the hydrodynamic radius R_*H*_ of solute *i*. The translational and rotational diffusion coefficients $D_{i}^{t}$ and $D_{i}^{r}$ of solute *i* are then scaled by a function that depends on its local occupied volume fraction $V_{i}^{\mathrm{frac}}$. The scaling function used is that defined by Tokuyama (71) or the translational coefficient and by Cichocki (72) for the rotation coefficient (see *SI Appendix* for a detailed description of these two functions). The rescaled values $D_{i}^{t_{\mathrm{short}}}$ and $D_{i}^{r_{\mathrm{short}}}$ are then used to propagate the motion of the solutes. The translational and rotational displacements are given by

[2] $$\begin{array}{c} \Delta r_{i}=\frac{D_{i}^{t_{\mathrm{short}}}\left(V_{i}^{\mathrm{frac}}\right)}{k_{B}T}F_{i}\Delta t+R_{i} \end{array}$$

[3] $$\begin{array}{c} \Delta w_{i}=\frac{D_{i}^{t_{\mathrm{short}}}\left(V_{i}^{\mathrm{frac}}\right)}{k_{B}T}T_{i}\Delta t+W_{i}, \end{array}$$

where $k_{B}$ and *T* are the Boltzmann constant and temperature, respectively, $r_{i}$ is the position of the center of geometry of solute i, $w_{i}$ is the orientation of solute i, $F_{i}$ is the force acting on solute *i*, $T_{i}$ is the torque acting on solute i, and $R_{i}$ and $W_{i}$ are the random translational and rotational displacements of solute *i*, which mimics the collision of solutes with solvent molecules. These displacements are defined by a Gaussian distributions with mean $\left\langle R_{i}\right\rangle =\left\langle W_{i}\right\rangle =0$ and variance $\left\langle R_{i}^{2}\right\rangle =6D_{i}^{t_{\mathrm{short}}}\Delta t$, $\left\langle W_{i}^{2}\right\rangle =6D_{i}^{r_{\mathrm{short}}}\Delta t$.

### Computation of the Bimolecular Association Rate Constant from BD Simulations in the Presence of Crowder Molecules.

The NAM algorithm (73) is used to calculate the bimolecular association rate constant for two solute molecules. The original algorithm defines two intermolecular distances at which the forces between the two solutes are centrosymmetric. The smaller distance *b* defines the so-called “b-surface,” while the larger distance *c* defines the “c-surface.” A trajectory is simulated by initializing one (usually the smaller) of the two molecules at a random position and orientation on the b-surface at the beginning of each trajectory to calculate the probability *β* that it will “react,” i.e. form a diffusional encounter complex, with the other molecule (fixed at the center of the two spherical surfaces) before reaching the c-surface. Once it reaches the c-surface, the trajectory finishes. Multiple trajectories are generated to compute *β* with the required statistical accuracy. To define the reaction between the two molecules, a criterion is defined based on the distances between pairs of close atoms in the bound complex (54). Once the probability *β* of “reacting” before “escaping” has been obtained, the association rate constant in infinitely dilute solution, $k_{\text{on}}^{\infty }$ is calculated as

[4] $$\begin{array}{c} k_{\text{on}}^{\infty }=4\pi D^{t}b\frac{\beta }{1-\left(1-\beta \right)\frac{k_{0}^{\infty }\left(b\right)}{k_{0}^{\infty }\left(c\right)}}, \end{array}$$

where *D*^*t*^ is the sum of the two absolute translational diffusion coefficients of the associating solute molecules in an infinitely dilute solution, and $k_{0}^{\infty }\left(b\right)$ and $k_{0}^{\infty }\left(c\right)$ are the rates at which the molecules reach the intermolecular distance separations, *b* and *c*, respectively. Assuming negligible intermolecular forces at these distances, the rates $k_{0}^{\infty }\left(b\right)$ and $k_{0}^{\infty }\left(c\right)$ can be calculated using the Smoluchowski equation as $k_{0}^{\infty }\left(r\right)=4\pi D^{t}r$.

In a crowded solution, however, the presence of additional crowder molecules modifies the diffusive behavior of the associating solute molecules, altering their diffusion coefficients. To account for these effects, we incorporate the crowding-dependent diffusion coefficient into the calculation of the association rate constant:

[5] $$\begin{array}{c} k_{\text{on}}\left(\phi \right)=4\pi D^{t}\left(\phi \right)b\frac{\beta }{1-\left(1-\beta \right)\frac{k_{0}\left(b\right)}{k_{0}\left(c\right)}}, \end{array}$$

where $D^{t}\left(\phi \right)$ is the sum of the effective diffusion coefficients of the two associating molecules as determined from BD simulations at the given value of the total volume fraction occupied by the crowder molecules *ϕ*, and $\frac{k_{0}^{\infty }\left(b\right)}{k_{0}^{\infty }\left(c\right)}=\frac{k_{0}\left(b\right)}{k_{0}\left(c\right)}=\frac{b}{c}$. Comparing the association rate constants from Eqs. **4** and **5** allows us to quantify and isolate the impact of the crowder-induced effect on diffusion coefficients of the associating solutes on the association rate constant.

**Fig. 1.**
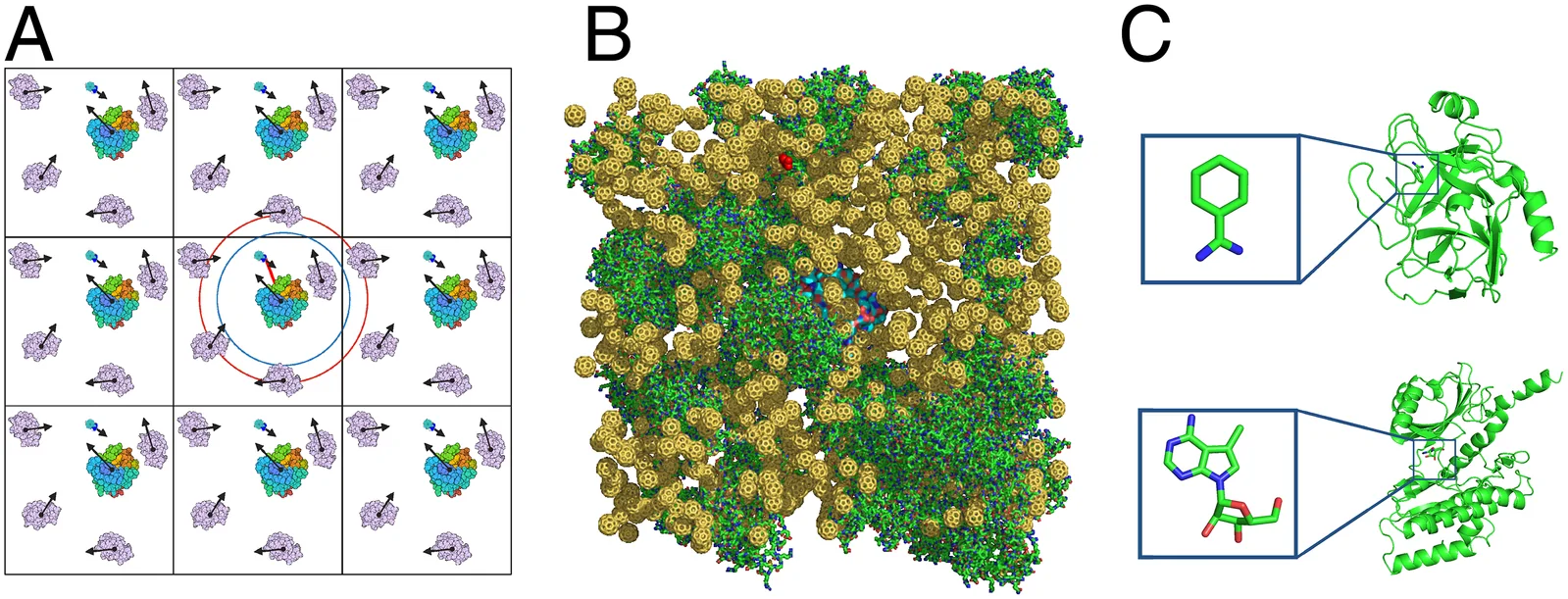
Illustration of the simulation set-up for computing the bimolecular association rate constant for a protein receptor–ligand complex in the presence of a solution of macromolecular crowders. (*A*) Schematic illustration of a snapshot of a simulated system in a periodic simulation box in which the NAM algorithm is used to compute the bimolecular association rate constant of a ligand and a protein receptor (both shown colored by the rainbow). The b- and c-surfaces (shown respectively in blue and red) can extend across periodic boundaries. The target receptor protein is at the center of the two surfaces, while crowder proteins are shown in violet. The arrows on the molecules indicate the directions of motion at the snapshot. (*B*) Representative snapshot taken from a BD simulation of the association of one trypsin and one benzamidine molecule in a periodic cubic simulation box with sides of length 200 Å in the presence of a crowder mixture of 59 lysozyme protein molecules (colored by atom type with green carbons) and 1,171 fullerene balls (gold color). The total volume fraction occupied by crowders, *ϕ* = 0.174. This snapshot is illustrative of the simulation set-up only; see *SI Appendix*, Fig. S13 for a quantitative analysis showing that there is no phase separation or self-aggregation of crowders. Trypsin at the center of the box is shown in surface representation (colored by atom type with cyan carbons), while benzamidine is shown in red using a van der Waals sphere representation. (*C*) Structures of the bound protein–ligand complexes studied: trypsin-benzamidine (*Top*) and haspin-chlorotubercidin (*Bottom*). The proteins are depicted in green cartoon representation and the ligands are shown in the *Insets* in stick representation colored by atom-type with green carbons.

In the present study, we have modified the code of SDA 7 in order to implement the NAM algorithm for the case where the solvent contains many crowder solutes in addition to the two solutes whose association rate constant is computed. The simulated system consists of a box with periodic boundaries with sides longer than the diameter of the c-surface sphere. The distance between the two solutes whose association rate is to be calculated is monitored at each time step, and one of the two molecules is relocated to a random position and orientation on the b-surface to begin a new trajectory as soon as the distance between the two solutes reaches the c-surface (Fig. 1*A*). Meanwhile, all the crowder solutes are free to diffuse in the box during the simulations.

### Intermolecular Energies and Forces.

The intermolecular forces are determined as the gradient of the pairwise interaction free energy. The energy terms used are those already implemented in SDA 7, with the exception of the interactions between crowder solutes, which are described in the following subsection. For each pair of solutes, the interaction free energy $\Delta G^{1-2}$ is given by the sum of energy terms, which are precomputed and stored in 3-dimensional arrays:

[6] $$\begin{array}{c} \begin{array}{cc} \Delta G^{1-2}= & \Delta G_{\mathrm{el}}^{1-2}+\Delta G_{\mathrm{edesolv}}^{1-2}+\\ & +\Delta G_{\mathrm{np}}^{1-2}+\Delta G_{\mathrm{ljrep}}^{1-2} \end{array} \end{array}$$

A detailed description of each energy term can be found in ref. 53. In brief, $\Delta G_{\mathrm{el}}^{1-2}$ describes the electrostatic interactions computed with the effective charge model (74), $\Delta G_{\mathrm{edesolv}}^{1-2}$ accounts for the unfavorable contribution to the interaction energy due to charge desolvation when two solutes approach each other (75), $\Delta G_{\mathrm{np}}^{1-2}$ describes the favorable contribution to the interaction energy resulting from the reduction of the total solute–solvent interface when two solutes approach each other (76), and finally $\Delta G_{\mathrm{ljrep}}^{1-2}$ is a Lennard-Jones (LJ) repulsive term that prevents solutes from overlapping (67).

### Adaptive Model for Crowders.

The calculation of the interaction energy between many crowder solutes with Eq. **6** can easily result in excessive computational costs. To solve this problem, we implemented an adaptive resolution model for crowders, which speeds up calculations (*SI Appendix*, Fig. S1) while giving similar results to the standard model (*SI Appendix*, Fig. S2). When the crowders are close to the two associating molecules (the noncrowders), the crowder interactions with the noncrowders are computed using Eq. **6**. However, when the crowders are beyond a threshold distance from the noncrowders, the crowder interactions are computed considering the crowder solutes as charged spheres with a radius equal to their radius of gyration. To avoid discontinuities between the two representations, we ensure a smooth transition in the electrostatic term, which is the main long-range contributor to such potential artifacts. As proposed by Mereghetti et al.(77), when a crowder lies partly inside and partly outside an electrostatic grid, the electrostatic forces are evaluated in both the effective-charge and Debye–Hückel descriptions, and the resulting force is taken as a weighted sum of the two, with weights proportional to the fraction of the solute in each region. The threshold distance, which is computed between the centers of geometry of the respective solutes, is set at half the length of the longest side of the largest interaction grid of any solute, be it crowder or noncrowder. The coarse-grained sphere model of the crowders is used to compute all crowder–crowder interactions. In the adaptive resolution model, the coarse-grained electrostatic interactions between the crowders are calculated solely with the Debye–Hückel model (77) while a Lennard-Jones repulsive term prevents steric clashes:

[7] $$\begin{array}{c} \Delta G_{\mathrm{adaptive}}^{1-2}=\Delta G_{\mathrm{DH}}^{1-2}\left(r\right)+\Delta G_{LJ_{\mathrm{adaptive}}}^{1-2}\left(r\right), \end{array}$$

where $\Delta G_{\mathrm{DH}}^{1-2}\left(r\right)$ corresponds to the same Debye–Hückel potential as used for the analytical correction to the electrostatic interaction energy of all solutes in the far-field due to the use of a finite grid size for computing $\Delta G_{\mathrm{el}}^{1-2}$ (77, 78), and the $\Delta G_{LJ_{\mathrm{adaptive}}}^{1-2}$ term between two solutes is defined solely by their volumetric radii *a*_1_ and *a*_2_,

[8] $$\begin{array}{c} \Delta G_{LJ_{\mathrm{adaptive}}}^{1-2}\left(r\right)=[\frac{a_{1}+a_{2}}{r-\gamma \;\cdot \;(a_{1}+a_{2})}{]}^{6}, \end{array}$$

where *r* is the distance between the two solutes and *γ* is a parameter that controls the effective range of the interaction. This adaptive Lennard-Jones term is from Tokuyama (71), where it was shown that a value of *γ* = 0.25 gives good agreement with experimental data in BD simulations of hard spherical colloids.

## Materials and Methods

The crystallographic structures of the proteins and ligands were downloaded from the Protein Data Bank (79) using the PDB codes given in *SI Appendix*, Table S1 (see Fig. 1*C* for visualization of the bound states). The structures were then protonated and assigned partial charges (80–82). Electrostatic potential grids and effective charges (74, 83), as well as grids for other interaction terms, were computed for each solute (for more details, refer to *SI Appendix*, Text and Table S2). The infinite-dilution translational and rotational diffusion coefficients of the molecules were calculated using the HYDROPRO software which estimates solution hydrodynamic properties (including diffusion coefficients and radii of gyration) directly from an atomic detail structure. The reaction criteria used to define encounter complexes were based on protein-ligand contacts identified in the crystal structure of the bound complex, and all independent contacts were required to be met (further details in *SI Appendix*).

Ligand binding pathways along BD trajectories were analyzed with Markov State Models (MSMs) (*SI Appendix*, Text and Fig. S4) and $k_{\mathrm{on}}$ values were calculated with the NAM algorithm (Eq. **5**). CAVER (84) was used to study channel formation by crowders and its impact on the binding mechanisms of the ligands (see *SI Appendix*, Text and Fig. S14 for more details).

## Results and Discussion

We first investigated the association of trypsin and benzamidine and of haspin and chlorotubercidin in the presence of varying concentrations of charged or neutralized HEWL crowders. Bimolecular association rate constants for the receptor–ligand complexes were computed using both the standard NAM algorithm (Eq. **4**) and the modified formulation that accounts for reduced translational diffusion rates of the solutes due to the presence of the crowders (Eq. **5**). We refer to the former as association rate constants without bulk translation diffusion coefficient correction (denoted as $k_{\mathrm{on}}\left(\phi ,D_{0}^{t}\right)$) and to the latter as association rate constants (denoted as $k_{\mathrm{on}}\left(\phi ,D^{t}\left(\phi \right)\right)$)–these are the rates that would be observed in the systems studied.

### Trypsin–Benzamidine Association Is Affected by Caging by Charged HEWL Protein Crowders.

**Fig. 2.**
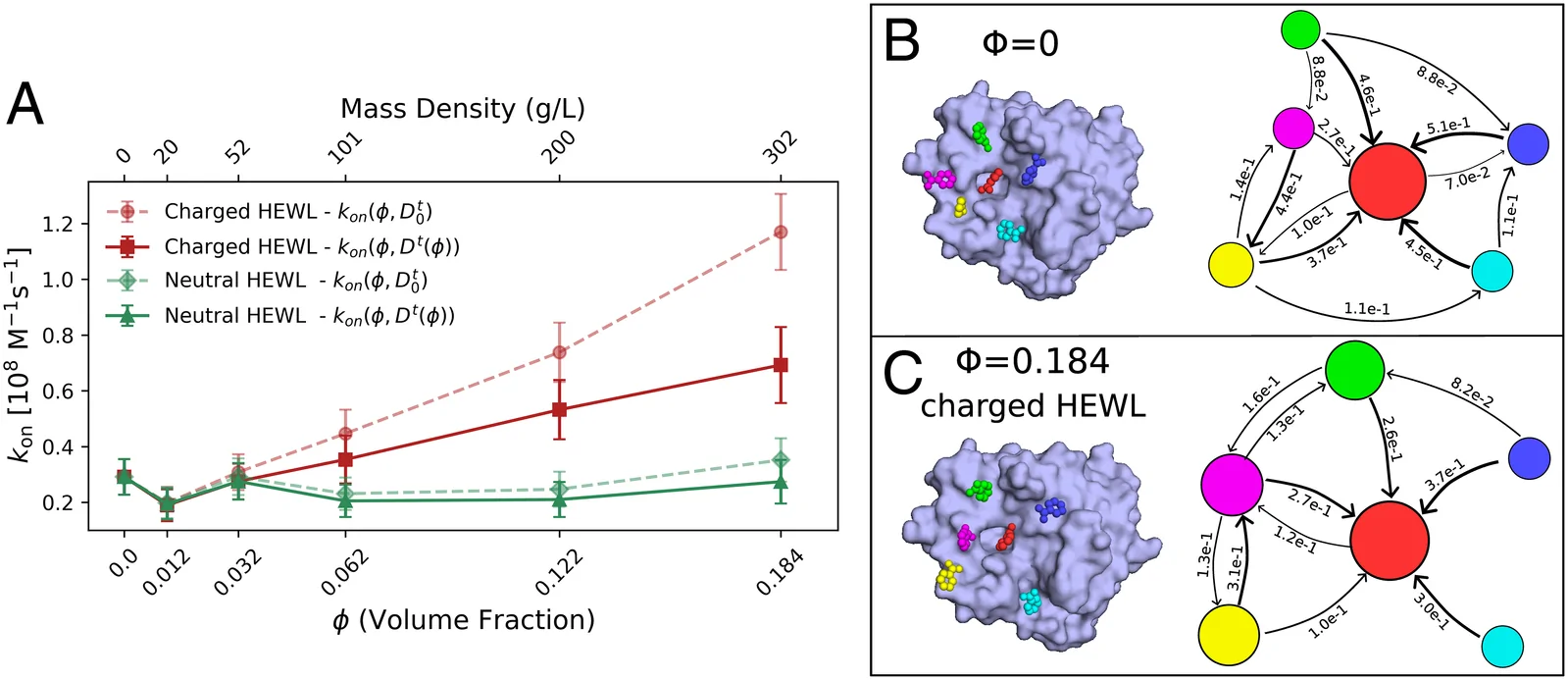
The effect of HEWL crowders on trypsin–benzamidine association is dependent on crowder density and charge with caging by charged HEWL crowders. (*A*) Values of the bimolecular association rate constant for trypsin and benzamidine computed at various HEWL crowder densities. Simulations were run with and without effective charges on the HEWL crowders. (*B*) Markov state model (MSM) of encounter trajectories in dilute solution (*ϕ* = 0, no crowders). The relatively larger size of the bound state cluster (red) compared to all other states indicates binding without long-lived secondary sites. (*C*) MSM of encounter trajectories in the presence of charged HEWL crowders at a volume fraction of *ϕ* = 0.184. The clusters are more dispersed over trypsin’s surface, revealing transient secondary binding sites (cyan and yellow states) and the ligand is observed to roll over the surface of trypsin and into the binding pocket due to crowder-induced caging (e.g. to the bound state from the yellow state via the pink state). In (*B* and *C*), the solvent accessible molecular surface of trypsin is shown with representative poses of benzamidine colored according to the respective states in the MSM, which is shown on the *Right* along with transition probabilities.

The results of the BD simulations show that, in the presence of charged HEWL crowders, increasing the crowder density leads to a systematic increase in the trypsin–benzamidine association rate constant, see Fig. 2. Residue-level contact-frequency mapping (*SI Appendix*, Fig. S9) shows that this enhancement does not arise from a uniform shell of crowders around trypsin, but from a specific, asymmetric electrostatic effect. A cluster of positively charged trypsin residues on the face opposite the binding pocket (Lys27, Lys109, Lys169, Lys188, Lys204, and Lys222) repels the positively charged HEWL crowders (net charge: +8e) away from that face, concentrating them instead in the vicinity of the binding pocket. Once benzamidine (net charge: +1e) diffuses into this crowder-dense region, its lateral escape along the trypsin surface is restricted by electrostatic repulsion from positively charged HEWL surface residues, notably Arg21 (*SI Appendix*, Fig. S9). This local confinement traps the ligand close to the trypsin surface and promotes encounter complex formation at the binding site. This behavior is further supported by the MSMs constructed from encounter trajectories under dilute and crowded conditions (Fig. 2, *Right*). In both cases, the bound state cluster (colored red) acts as a probability sink. However, at higher crowder density (*ϕ* = 0.184), the intermediate clusters are more dispersed over the trypsin surface, indicating that benzamidine explores secondary binding sites before forming the bound state. An illustrative path is initial binding to the yellow outer cluster, transition through the intermediate state magenta cluster, and ultimately reaching the native bound configuration (red cluster). In contrast, under dilute conditions (*ϕ* = 0), the pathway to binding is more direct, with fewer transitions through metastable states. The trend of increasing association rate with increasing crowder density is more prominent for $k_{\mathrm{on}}\left(\phi ,D_{0}^{t}\right)$ but is preserved for the association rate constant (Fig. 2*A*). The monotonic rise in the association rate constant indicates that, at concentrations representative of physiological intracellular environments (85), electrostatic caging effectively outweighs the diffusion-damping effects induced by macromolecular crowding.

In contrast, simulations with neutralized HEWL crowders (with all charges set to zero) do not exhibit such enhancement in association rate constant with increasing crowder concentration. This is primarily due to the change in crowder-trypsin and crowder–benzamidine interactions (*SI Appendix*, Figs. S9 and S11) as switching off the charges has little effect on the crowder–crowder interactions (*SI Appendix*, Fig. S12) and radial distribution functions (*SI Appendix*, Fig. S10), and indicates that the caging effect is an electrostatic phenomenon. Moreover, the effect of reduction of the solute diffusion coefficients in the neutral HEWL crowders simulations is primarily because benzamidine experiences less reduction in its translational diffusion coefficient in the absence of electrostatic interactions with the crowders (*SI Appendix*, Fig. S3). Furthermore, the MSM for neutralized crowders (*SI Appendix*, Fig. S5) is rather similar to that in dilute solution, indicating that the binding mechanism is unaffected by the addition of neutralized crowders.

### Haspin–Chlorotubercidin Association Is Affected by Channeling by Charged HEWL Protein Crowders.

**Fig. 3.**
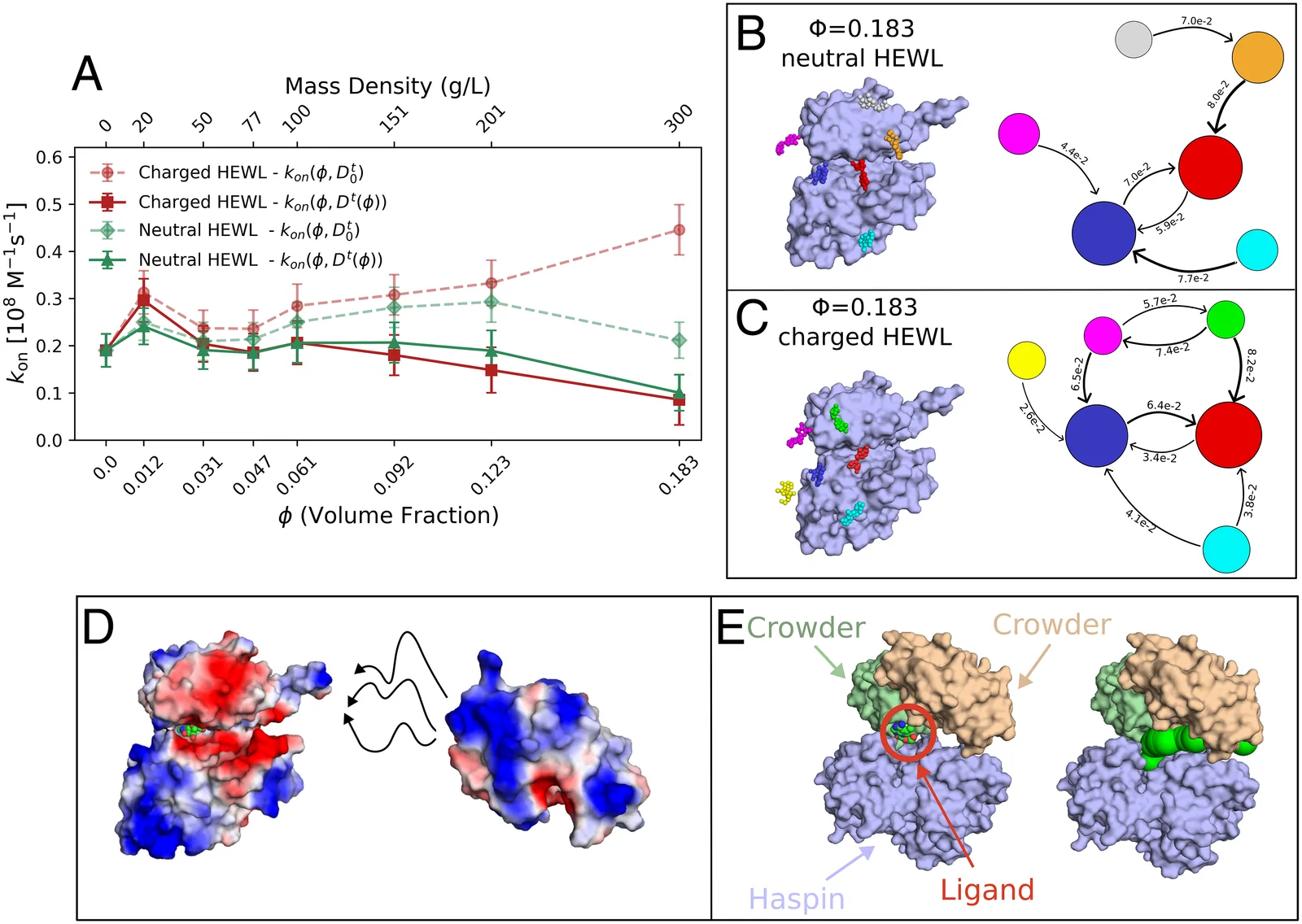
The effect of HEWL crowders on haspin–chlorotubercidin association is dependent on crowder density and charge, with channeling by charged HEWL crowders. (*A*) Values of the bimolecular association rate constant for haspin and chlorotubercidin computed at various charged and neutral HEWL crowder densities. (*B*) MSM of encounter trajectories in the presence of neutralized HEWL crowders at a volume fraction of *ϕ* = 0.183. Chlorotubercidin can bind to the gray state and reach the binding pocket (red state) through the orange state; alternatively chlorotuberdicin can also bind to either magenta or cyan clusters and then transition to the blue cluster before reaching the red bound state. (*C*) MSM of encounter trajectories at the same crowder density computed for charged crowders. The gray cluster above the binding pocket disappears due to attractive electrostatic interactions between HEWL and this surface region. A new cluster (green), appears from which the ligand transitions to either the magenta or the bound (red) states. The yellow state, also absent in the simulations with neutralized HEWLs, allows transitions to the blue cluster to subsequently reach the binding pocket. (*D*) Electrostatic potentials of haspin (*Left*) and HEWL (*Right*) shown mapped onto their molecular surfaces. Chlorotuberdicin (van der Waals spheres colored by atom type) is shown bound to haspin’s active site, which is surrounded by a highly negatively charged (red) region that attracts the positively charged (blue) region of HEWL (which has a net charge of +8e). This interaction explains the absence of MSM intermediate states above the binding pocket when charged HEWL crowders are present. (*E*) Channeling effect due to electrostatic interactions between haspin (light blue) and HEWL crowders (light orange and green). *Left*: a frame from a late-stage encounter trajectory with charged HEWL crowders. *Right*: a channel originating at the haspin binding pocket entrance (green spheres). The channel was identified by using CAVER with average bottleneck radius of 4.73 Å, maximum bottleneck radius of 4.71 Å, and average tunnel length of 52.10 Å.

Compared with benzamidine, chlorotubercidin is a significantly larger molecule. As a result of its size, its diffusion is more strongly damped by crowders than that of benzamidine (*SI Appendix*, Fig. S3). However, the association rates are maintained at low crowder concentration and only modestly damped at higher crowder concentration (Fig. 3*A*). Particularly in the case of charged HEWL crowders, there is an association-enhancing mechanism that competes with the excluded volume effect. This enhancement arises from electrostatic interactions between a negatively charged surface patch near haspin’s active site and the positively charged HEWL (Fig. 3*D*). Notably, the interaction of crowders with this attractive patch on haspin does not occlude the binding pocket but rather localizes HEWL crowders in a region that promotes ligand retention near the pocket entrance. A lysozyme crowder occupying this site can hinder chlorotuberdicin from diffusing away along the protein surface, effectively facilitating productive encounters.

To explore this mechanism, we constructed MSMs of the binding pathways in the presence of both charged and neutralized HEWL crowders at *ϕ* = 0.183 where the difference in the computed association rate constants without the bulk translation diffusion coefficient correction is clearly recognizable. A key distinction between the MSMs lies in the spatial distribution of the intermediate states: With the neutral crowders, several ligand clusters reside near the negatively charged patch on haspin, with ligand sampling being more broadly dispersed. In contrast, simulations with charged crowders show a more focused ligand cluster distribution close to the binding site, with an enhanced population of microstates corresponding to the binding site (red and blue clusters, Fig. 3*C*). This difference arises from localized steric confinement: because charged HEWL crowders—in contrast to neutral HEWL crowders—are drawn closer to the binding pocket entrance by electrostatic attraction to haspin, they establish a physical barrier that prevents the neutral ligand from diffusing away back into the bulk. Simultaneously, these crowders form dynamic, transient channels that guide the neutral ligand directly into the active site. Further confirmation of this phenomenon was obtained by analyzing the final frames of encounter trajectories with CAVER, a tool for detecting channels and tunnels in protein structures. Using the ligand’s hydrodynamic radius as the minimum probe size and the binding pocket as the tunnel origin, we identified solvent-accessible pathways extending up to 60 Å between neighboring HEWL crowders (Fig. 3*E*). These channels support the hypothesis that, while crowder–crowder interactions are not affected by switching on charges (*SI Appendix*, Fig. S10), the protein–crowder electrostatic interaction forms a dynamic funneling network that enhances ligand diffusion to the binding site. In the absence of these interactions, i.e. with neutralized HEWL crowders, this effect is lost which is the reason why the $k_{\mathrm{on}}\left(\phi ,D_{0}^{t}\right)$ values are more similar to the $k_{\mathrm{on}}\left(\phi ,D^{t}\left(\phi \right)\right)$ values.

### Association Rate Constants Have a Nonmonotonic Dependence on the Volume Fraction of C60 Fullerene Crowders.

**Fig. 4.**
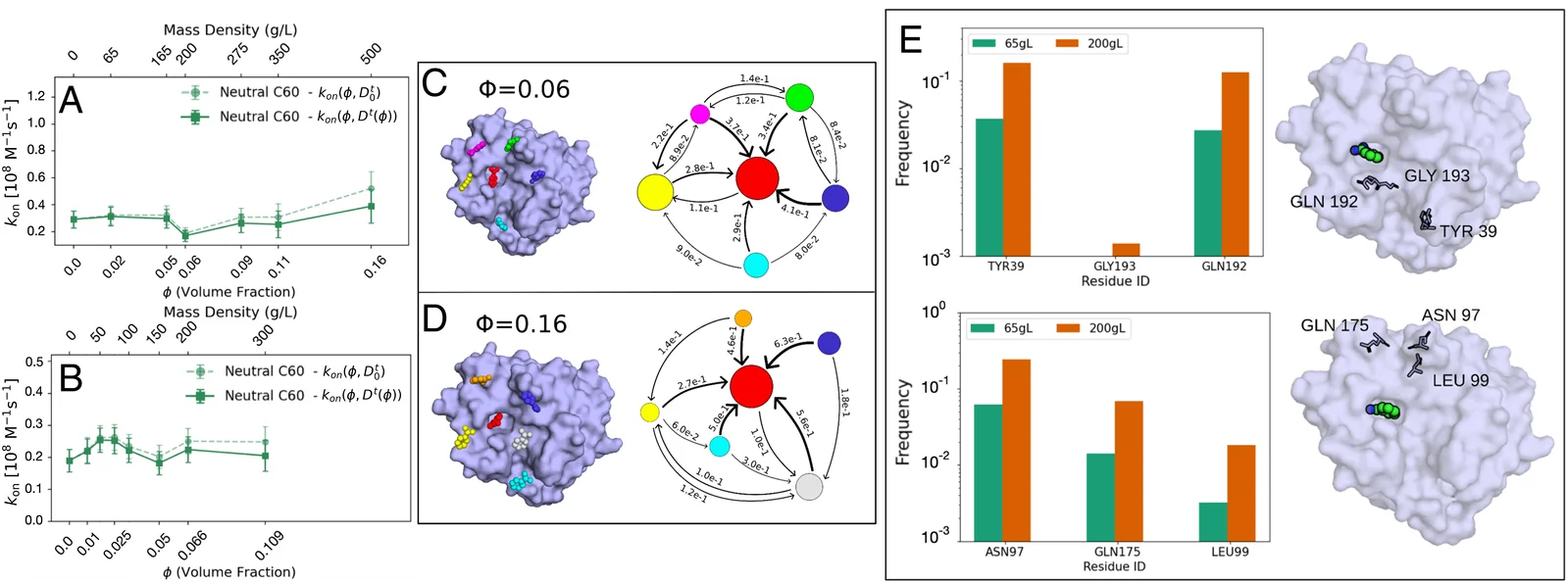
${\text{C}}_{60}$ fullerene crowders result in a nonmonotonic dependence of association rate constants on crowder concentration. (*A*) Computed values of bimolecular association rate constants for benzamidine binding to trypsin at varying ${\text{C}}_{60}$ fullerene crowder densities. A nonmonotonic trend emerges, with an inversion in the trend at *ϕ* = 0.06. (*B*) Computed values of bimolecular association rate constants for chlorotubercidin binding to haspin at various ${\text{C}}_{60}$ fullerene crowder densities. (*C* and *D*) MSMs for trypsin–benzamidine association at *ϕ* = 0.06 and *ϕ* = 0.16, respectively. (*E*) Two concave regions near the trypsin binding pocket, lined by residues GLN192, GLY 19, and TYR39 (Upper plot and figure), and GLN17, ASN97, and LEU99 (*Lower* plot and figure), that accommodate C60 fullerene crowders–cf. (*SI Appendix*, Fig.S6). *Left*: Interaction frequency increases as crowder density increases. *Right*:Positioning of the two concave regions on the surface of trypsin with benzamidine shown as van der Waals spheres in the binding pocket. At *ϕ* = 0.06 (*C*), the gray cluster seen at *ϕ* = 0.16 at the concave region near residues GLN192, GLY193, and TYR39 (*E*) is absent, and the magenta and green clusters are further away from the concave region lined by residues GLN17, ASN97, and LEU99 than the orange cluster at *ϕ* = 0.16: This is due to ${\text{C}}_{60}$s occupying concave surface regions and obstructing benzamidine diffusion along the surface. At *ϕ* = 0.16 (*D*), the high density of fullerenes creates a caging effect around trypsin that enhances benzamidine diffusion along the protein surface, restoring the positions of the orange and gray clusters close to the concave regions described above: prolonged residence of benzamidine on the surface (due to the caging effect) increases the probability that fullerenes dissociate, making room for ligand binding.

To investigate ligand–protein association in the presence of smaller crowders, we simulated the association of the trypsin–benzamidine and haspin–chlorotubercidin pairs in the presence of ${\text{C}}_{60}$ fullerene crowders. In addition to serving as a model for small molecular species within the cellular environment, fullerenes have a rigid, nearly spherical geometry that makes them particularly well suited for BD simulations under the rigid-body approximation. Moreover, their small size has enabled extensive study by molecular dynamics (MD) simulation, providing useful benchmarks for comparison. Indeed, a nonmonotonic dependence of the association rate constant on C60-like crowder concentration has been previously reported in coarse-grained MD (51) and BD (48) studies. Consistently, we observe a similar nonmonotonic trend in the computed values of the association rate constant for the trypsin–benzamidine system with increasing fullerene crowder density (Fig. 4*A*). Specifically, the association rate constants initially decrease, reaching a minimum at a crowder volume fraction of *ϕ* = 0.06, and then steadily increase up to *ϕ* = 0.16. While this trend is qualitatively robust, the overall magnitude of the variation in $k_{\mathrm{on}}$ is relatively modest. The small size of this variation is in agreement with BD simulations with spherical crowders (48) suggesting that, while the physical consequences of the competing diffusion and caging effects are present, it would be difficult to isolate and detect them experimentally.

To elucidate the underlying mechanism, we analyzed encounter trajectories using MSMs. At higher crowder densities (Fig. 4*D*), the transition probability from intermediate clusters to the bound state (red cluster) is significantly higher compared to lower crowder densities (Fig. 4*C*). This indicates that the high local concentration of fullerenes enhances the likelihood of surface diffusion events that guide the ligand toward the binding pocket: a steric retention of the ligand on the protein surface governed by the presence of fullerenes which act as physical barriers that increase the residence time of the ligand near the protein surface. However, it is important to note that this excluded volume caging effect is less pronounced than that observed in the presence of charged HEWL crowders, where long-range electrostatic repulsion plays a dominant role in confining the ligand even at smaller crowder densities. Nevertheless, our results demonstrate that under high crowding conditions—up to concentrations approaching 500 g/L— even small, neutral crowders can enhance association kinetics via steric confinement alone.

The observed decrease in association rates around a crowder volume fraction of *ϕ* = 0.06 can be attributed to specific steric interactions between fullerenes and the trypsin surface. In particular, two concave regions located between residues 39, 192, and 193 (between the cyan and red clusters) and between residues 97, 99, and 175 (near the green and blue clusters) appear especially well-suited to accommodate the spherical geometry of ${\text{C}}_{60}$ fullerenes (Fig. 4 *C* and *E*). Analysis of crowder–residue contact probabilities reveals that interactions at these sites become increasingly likely as fullerene density increases. This shows that at *ϕ* = 0.06 density, the fullerene concentration is sufficient to occupy these concave regions with high probability, but still insufficient to induce the global bulk caging effect responsible for enhancing ligand retention at higher densities. Occupation of these sites by fullerenes effectively obstructs key ligand diffusion pathways that pass through the green and blue intermediate states (Fig. 4*C*). As a result, surface diffusion of benzamidine is hindered, leading to a reduction in association rate constants. This suggests that at moderate crowding levels, crowders can impose local steric constraints that interfere with productive binding pathways, even in the absence of strong long-range interactions.

The impact of fullerenes on the association between haspin and chlorotubercidin at high densities is less pronounced compared to the trypsin–benzamidine system Fig. 4*B*, where the $k_{\mathrm{on}}$ increases. A slight reduction in association rate constants is observed around a crowder volume fraction of *ϕ* = 0.05, which can be attributed to increased surface interactions between fullerenes and the protein. At this density, the system is sufficiently crowded to permit local steric interference but not dense enough to induce the bulk steric caging effect observed at higher crowder concentrations. In the trypsin–benzamidine system, the caging effect induced by excluded volume described above emerges at densities corresponding to approximately 500 g/L. However, in the case of haspin, the significantly larger size of the protein necessitates a proportionally larger simulation box, rendering such high crowder densities computationally infeasible, even with the simplified models used in the BD simulations. As a result, the high-density regime required to observe excluded volume enhancement of association rates remains unexplored for this system.

### Mixtures of Neutralized HEWL and C60 Fullerene Crowders Have Additive, Countering Effects on the Receptor–Ligand Association Rate Constants.

Cellular environments are inherently heterogeneous, containing a diverse array of macromolecular crowders that vary in size, shape, and type of interactions. To capture some of this complexity, we performed simulations using a binary mixture of neutralized lysozymes and ${\text{C}}_{60}$ fullerenes for a range of total crowder densities. The two crowder types were combined such that their total masses were equal, thereby maintaining a consistent contribution to the excluded volume. Given that HEWL has a molecular mass of approximately 14 kDa and C60 fullerene has a mass of 0.72 kDa, the particle number ratio used in the simulations was 1:20 (HEWL:fullerene).

**Fig. 5.**
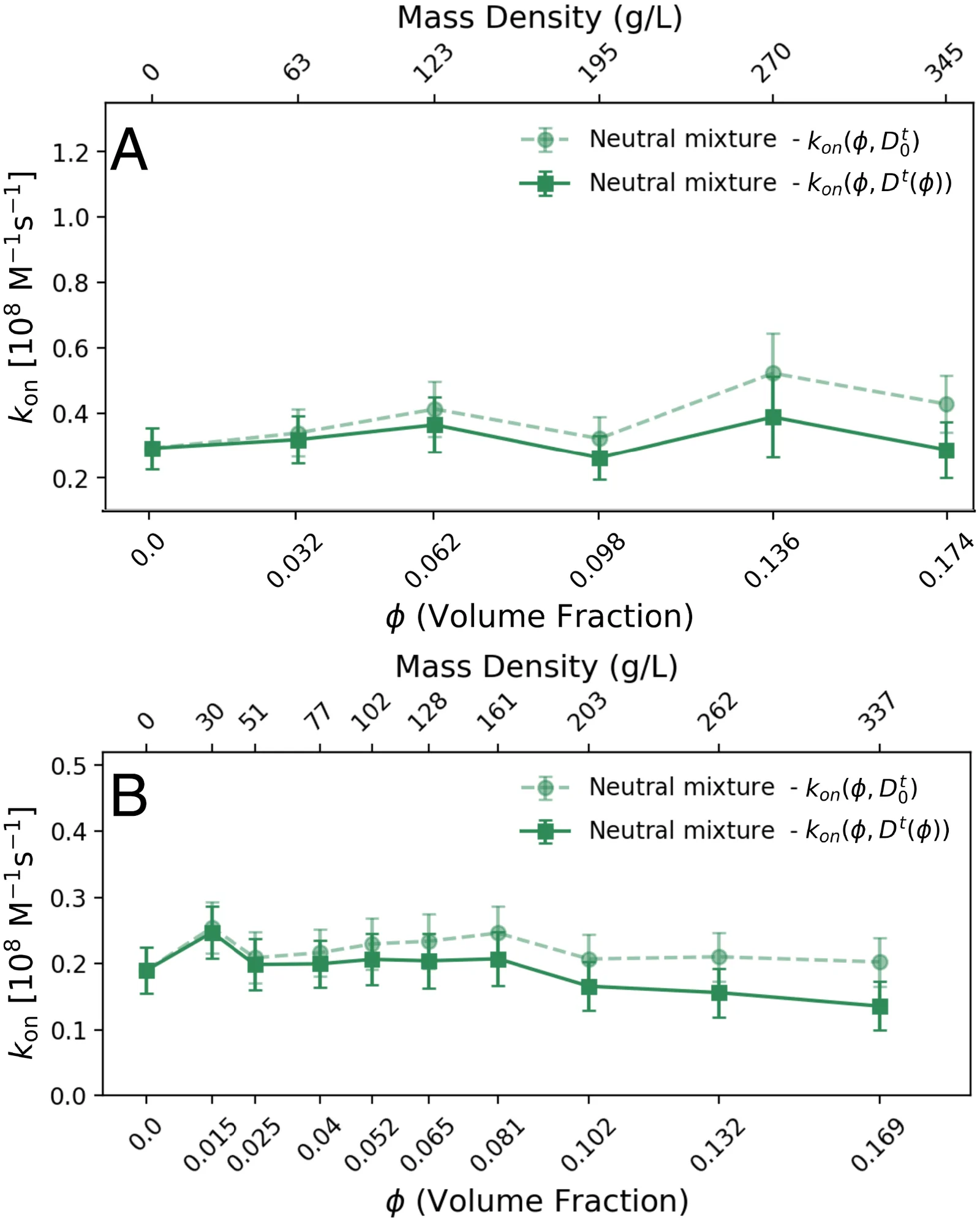
Computed values of association rate constants in mixtures of C60 fullerene and neutralized HEWL crowders for (*A*) trypsin–benzamidine and (*B*) haspin–chlorotubercidin complexes. The crowder mixture maintains a lysozyme:C60 particle ratio of 1:20 to ensure the same total excluded volume for each of the two crowder types. In both systems, the trends suggest an additive effect of the mixture on the computed values, closely resembling the behaviors observed in the single-crowder simulations. At the same time, the presence of both crowder types dampens crowder-specific effects, leading to a more averaged response in the mixed-crowder environment.

Interestingly, in both receptor–ligand systems, the mixed-crowder environment appears to exert an additive influence on the specific trends observed in the single-crowder simulations. This effect is particularly evident in the trypsin–benzamidine system (Fig. 5*A*). A drop in the association rate constant is observed around a crowder volume fraction of *ϕ* = 0.098–as in the simulations with only fullerene crowders—but, unlike the fullerene-only case, there is no subsequent increase in association rates at higher densities. Instead, the $k_{\mathrm{on}}$ values plateau, reflecting the stabilizing behavior seen at high densities in simulations with neutral HEWL crowders. Overall, while the qualitative trends observed with individual crowder types are still discernible in the mixed system, they are notably attenuated. This suggests that the mixture not only produces an additive effect on the association kinetics but also acts to average out specific behaviors observed in single-crowder simulations. As a result, crowder-specific effects are preserved but less pronounced in the mixed-crowder environment.

For the haspin–chlorotubercidin pair, the computed values of association rate constant remain largely unaffected by the presence of either neutral HEWL or fullerene crowders alone, and this trend persists in the mixed-crowder simulations (Fig. 5). Across all tested crowder densities, the association rates remain comparable to those observed under dilute conditions, indicating that in systems where binding is governed predominantly by hydrophobic interactions, excluded volume effects alone do not significantly alter association kinetics. These insights from the haspin–chlorotubercidin system reinforce the dual role of the mixed-crowder environment: reproducing in an additive fashion the individual effects observed in single-crowder systems and mitigating their variability, resulting in a more uniform association behavior.

## Conclusions

We have introduced an adaptive dual-resolution model to simulate protein-small molecule association in the presence of different macromolecular crowders. Crowder molecules are simulated at atomistic resolution when they are in proximity to the specifically binding protein and ligand, while coarse-grained resolution is imposed on crowder—noncrowder interactions for crowders that are beyond a threshold distance from the noncrowders and on crowder—crowder interactions regardless of their proximity. The computational advantage of this hybrid model is that it makes it possible to simulate the association process of different systems, with different crowding agents, at different densities extending up to the high crowding density inside bacterial cells.

We performed extensive BD simulations for two receptor–ligand complexes with very different physicochemical properties, trypsin–benzamidine and haspin–chlorotuberdicin, to study the impact of two different crowders (the protein HEWL and the C60 fullerene) and their interaction properties on the association rates of the complexes.

In simulations where only HEWL crowders were present, we observed that charged lysozyme crowders provide an enhancement mechanism that competes with the damping in the diffusion which, in contrast, tends to decrease the association rates. However, the mechanism that led to this increase is different in the two cases. For the trypsin-benzamidine system, the enhancement in k_*on*_ results from the formation of a Faraday-like box, which was confirmed with MSM analysis. In dilute conditions, ligand clusters around the trypsin surface are small in population compared to the cluster formed at the entrance of the binding site. However, at high densities, the ligand clusters around the trypsin surface become bigger when the charged lysozyme is employed as the crowder in the simulations (Fig. 2). This suggests a steric retention mechanism that promotes surface exploration and increases binding probability.

For the haspin-chlorotuberdicin system, electrostatic attraction between the positively charged HEWL crowders and a negative surface patch near the active site drives enhanced association through a dual mechanism. First, the localized accumulation of crowders provides steric confinement that prevents the ligand from escaping the active site region; second, this high crowder density creates transient channels that steer the neutral ligand from the bulk directly toward the binding pocket. CAVER analysis confirms the formation of these channels while MSM analysis shows that encounter complexes concentrate near the hinge region of haspin. Importantly, such channeling is not observed in the trypsin-benzamidine system (*SI Appendix*, Fig. S7), where the ligand explores the surface rather than associating via a crowder-guided process. This absence of channel formation is consistent with the lack of a well-defined electrostatic patch surrounding the trypsin binding pocket (*SI Appendix*, Fig. S8), in contrast to the pronounced electrostatic features present around the haspin active site.

Simulations with fullerene C60 crowders show a different trend. At moderate densities, fullerenes preferentially adsorb onto the trypsin surface, displacing benzamidine and reducing association rates. However, at higher densities, a steric retention mechanism emerges, partially reversing the reduction. Given the rigid-body approximation of these simulations, the accommodation of fullerene when adsorbing on the trypsin surface may be underestimated. To better analyze this mechanism, it is important to conduct simulations that incorporate molecular flexibility. Within the studied density range (0 to 350 g/L) for the haspin-chlorotuberdicin system, a nonmonotonic trend was observed, consistent with simulations of HEWL in the absence of charges, suggesting a balance between steric retention close to the protein binding pocket and damping in association due to steric hindrance effects.

Finally, simulations with a mixture of HEWL and fullerene crowders revealed an additive effect on association rates, while simultaneously averaging out crowder specific behaviors, in agreement with observations from experimental studies (13). In the trypsin-benzamidine system, this manifested in a plateau at higher densities rather than the nonmonotonic increase seen with fullerenes alone. On the other hand, in the haspin-chlorotubercidin system, association rates remained largely unaffected across all conditions. The magnitude of the crowding effects varies considerably across systems. Although the individual opposing effects are larger, when combined, the greatest effect on the association rate constant in the systems studied is a systematic increase for trypsin–benzamidine with charged HEWL crowders (Fig. 2). For haspin–chlorotubercidin with charged HEWL, the effect of the presence of the crowders is modest, but this reflects a near-cancellation between two opposing mechanisms, electrostatically driven channeling toward the binding pocket and crowding-induced damping of translational diffusion (Fig. 3), rather than an absence of crowding effects. The effects of C60 fullerene crowders and mixtures of C60 and HEWL crowders on the association rate constant are smaller.

In conclusion, this work demonstrates that BD simulations incorporating different types of crowders and interaction forces reveal distinct effects on k_*on*_ rates, with different binding mechanisms implicated. Our findings highlight fundamental distinctions between general and system-specific crowding effects: While increasing solute size and structural complexity universally damp translational and rotational diffusion across different crowded media, the ultimate net impact of crowding on protein–ligand association kinetics is dictated by highly specific, interfacial interactions. This highlights the necessity of highly efficient, multiscale computational frameworks to screen and decipher the association landscapes of diverse drug-protein pairs. Considering the rigid nature of the C60 fullerene and the hard nature of HEWL (86), the low number of degrees of conformational freedom of benzamidine and chlorotubercidin, and the similarity of the apo and holo protein receptor structures, the key features of the interactions between crowder and noncrowder solutes that influence association rates can be expected to be captured by the BD rigid body model. However, in these simulations, the rigid body treatment prevents chlorotubercidin from fully entering the haspin binding pocket through its narrow entrance whereas benzamidine can enter the trypsin binding pocket. Therefore, to capture and interpret the association mechanisms in crowded environments in full, future studies should incorporate molecular flexibility to simulate induced-fit effects (87).

## Supplementary Material

Appendix 01 (PDF)

## Data Availability

The input and output files for this study are available on Zenodo (88). Coordinate files were obtained from the Protein Data Bank (www.rcsb.org) as described in *Materials and Methods*. The software used in this study is as follows and is available for free for academic use. All software is also available for commercial use but in some cases, there is a licensing fee: Schrödinger suite, Protein Preparation Wizard (version 2024/r3); PDB2PQR (version v2.0); GAMESS (version v2020.2); HYDROPRO (version v10); SDA (versions 7.3.4 and 7.4.0); APBS (version 1.1.0); AMBERTOOLS (version 20); VMD (version v1.9.4); and CAVER (version 3.0.3).
