# Supplementary material for: Multiscale Brownian dynamics simulations reveal effects of macromolecular crowding on protein–ligand association
Source: Proc Natl Acad Sci U S A. 2026 Oct 30;123(44):e2617950123. doi: 10.1073/pnas.2617950123 (PMC13732080; doi:10.1073/pnas.2617950123)
Supplement: Supplementary file 1 [file pnas.2617950123.sapp.pdf]

Supporting Information for

Multiscale Brownian dynamics simulations reveal effects of macromolecular crowding on protein-ligand association

Riccardo Beccaria, Abraham Muñiz-Chicharro and Rebecca C. Wade

Corresponding Author: Rebecca C. Wade.
E-mail: rebecca.wade@h-its.org

This PDF file includes:

Supporting text
Figs. S1 to S14
Tables S1 to S2
SI References

Supporting Information Text

Supporting Theory

The diffusion coefficients in simulations are scaled by a function that depends on its local occupied volume fraction V_i^{frac} defined in Eq. 1 of the main text. The scaling function used is that defined by Tokuyama for the translational coefficient and by Cichocki for the rotation coefficient. Once the local occupied volume fraction has been computed, it is utilized at each Brownian dynamics simulation time step to compute the short-time translational ($D_i^{t_{short}}(V_i^{frac})$) and rotational ($D_i^{r_{short}}(V_i^{frac})$) self-diffusion coefficients. To account for short-time translational hydrodynamic interactions in a concentrated suspension of interacting particles, we employ the theoretical model derived by Tokuyama(1)

$$D_i^{t_{short}}(V_i^{frac}) = D_i^t / [1 + H(V_i^{frac})]$$

where D_i^t represents the baseline translational diffusion coefficient at infinite dilution (computed with HYDROPRO), and $H(V_i^{frac})$ describes the contributions originating from short-time static hydrodynamic interactions. The scalar function $H(V_i^{frac})$ is evaluated as:

$$H(V_i^{frac}) = \frac{2b^2}{1-b} - \frac{c}{1+2c} - \frac{bc(2+c)}{(1+c)(1-b+c)}$$

where $b = [\frac{9}{8} \cdot V_i^{frac}]^{0.5}$ and $c = \frac{11}{16} \cdot V_i^{frac}$.

Short-time rotational hydrodynamic interactions, including near-field lubrication forces alongside two- and three-body expansions of the mobility functions, are incorporated by scaling the rotational diffusion coefficient using the framework established by Cichocki et al.(2)

$$D_i^{r_{short}}(V_i^{frac}) = D_i^r \cdot [1 - 0.631 \cdot V_i^{frac} - 0.726 \cdot V_i^{frac^2}]$$

where D_i^r is the isotropic rotational diffusion coefficient at infinite dilution, which is calculated directly from the protein structure using HYDROPRO. These concentration-dependent diffusion coefficients are updated at every single integration step of the multi-scale BD simulations.

Supporting Materials and Methods

Protein receptor and ligand structure preparation. The crystallographic structures of the proteins were downloaded from the Protein Data Bank (3). For the trypsin and haspin proteins, both the unbound structure (apo-protein) and the structure in complex with the respective ligand (holo-protein) were used. Table S1 shows the pdb codes of the respective structures.

All protein structures were completed using the Schroedinger Suite Wizard module (version 22, release 4) (4) to add disulfide bonds, terminal oxygen (OXT) atoms and missing loops where absent. The protein structures were then protonated and atomic partial charges added using the pdb2pqr (version 2.1.1) software (5). Hydrogen atoms were added using the PROPKA method at pH 7, while the atomic partial charges were assigned according to the AMBER force field (6) at the same pH.

The ligand structures were extracted from the holo-structure of the complex with the protein. The ligand structures were first protonated by the PROPKA method at pH 7 using the Protein Preparation Wizard module of the Schroedinger suite (4), and then relaxed by performing energy minimization of the hydrogen atoms using the OPLS4 force field (7) (also within the Schroedinger suite). To obtain the atomic partial charges of the ligands, the protocol developed by (8, 9) was followed. First, the molecular electrostatic potential of the ligand is obtained by quantum mechanical calculations using the GAMESS software (10). Then, the partial charges of the ligand are obtained by fitting the molecular electrostatic potential using the Ambertools RESP module (v.20). Finally, the charges are redistributed over the polar atoms of the ligand and the charged residues of the proteins following the effective charge model of SDA.

Crowder preparation.. To simulate a crowded environment, two types of molecules were chosen as crowders, HEWL and the C60 fullerene. HEWL is a protein found in mucosal secretions with functions related to the immune system's response to bacteria. HEWL, being a protein, mimics the cellular environment better than fullerene, and its relatively small size and stable structure make it suitable for Brownian dynamics simulations. The structure was extracted from the PDB (see Table S1). The C60 fullerene, whose spherical shape, rigidity and small size (diameter of about 7Å) make it one of the most suitable molecules to simulate the association process in crowded environments, was modelled based on the structure retrieved from the Ligands section of the RCSB Protein Data Bank (entry 60C). It has previously been investigated as a crowder by coarse-grained molecular dynamics simulation (11).

Both the C60 fullerene and HEWL were prepared in the same way as described above for the receptor proteins. Whereas the C60 fullerene is neutral with all carbon atoms assigned partial atomic charges of zero, HEWL was computed to have a net charge of +8e at pH 7. Therefore, computations were also performed with all charges on HEWL neutralized in order to separately investigate the effect of crowding by non-specific protein-like molecules.

Brownian Dynamics Simulations. Precomputed interaction potential grids were used in the BD simulations to calculate the forces according to Eq.(6). The APBS software (12) was used to calculate the molecular electrostatic potential grids, which were cubic grids with a grid spacing of 1 Å , using the finite difference method to solve the linearized Poisson-Boltzmann equation. The temperatures (298K) and ionic strengths (100mM for trypsin-benzamidin, 150mM for haspin-chlorotubercidin) were set to the same values as used in the experimental measurements of the association rate constants (13, 14). For the boundary conditions, the single Debye-Hückel sphere approximation was used, meaning that the potential at the boundary of the grid is approximated by the potential generated by a corresponding point charge equal to the net charge of the solute molecule. Values of $\epsilon_s = 78$ and $\epsilon_p = 2$ were used for the permittivity of aqueous solution and the solute, respectively.

Effective charges were computed for use in the BD simulations(15). For the proteins, the standard SDA protocol was used to derive the effective charges, while for the small molecules, the protocol developed by (8) was used, where charges were assigned only to polar and halogen atoms.

For electrostatic desolvation, hydrophobic desolvation and Lennard-Jones repulsion interactions, precomputed grids were computed using the make_edhdlj_grid tool implemented in SDA 7.3.5. For the calculation of electrostatic desolvation interactions, a scaling factor of 0.36 and an ionic strength of zero were used (16). The standard hydrophobic desolvation model was modified as described by Muniz-Chicharro et al. (9) to exclude any hydrophobic desolvation contribution from atoms containing a formal charge (i.e.: the hydrophobic desolvation potential and the solvent-accessible surface area (SASA) associated with these atoms were both set to zero). Hydropro (17) was used to calculate the translational and rotational diffusion coefficients of the solutes at infinite dilution in aqueous solution, with a radius of the atomic element (AER) of 2.9 Å for proteins and 1.2 Å for small molecules (18). Details about the size of each interaction grid for each solute are provided in Table S2. To simulate crowded systems, periodic simulation boxes containing the solutes and crowders were generated at the required value of ϕ. ϕ was calculated by dividing the volume occupied by the crowders by the volume of the simulation box. To calculate the crowder volume, crowders were assumed to have a spherical shape with radius equal to their radius of gyration (4.8 Å for fullerenes and 15.0 Å for HEWL). The density of the simulation system in g/L units was computed as the total mass of the crowders was divided by the volume of the box.

For each system simulated, 200000 BD trajectories were run using b and c intermolecular distances for beginning and ending the simulations, respectively, of 80 Å and 100 Å for the trypsin-benzamidine system and of 80 Å and 120 Å for the haspin-chlorotuberdicin system. The simulations were performed in a cubic periodic box with sides of length 200 Å for the trypsin-benzamidine system and sides of length 240Å for the haspin-chlorotuberdicin system. A timestep of 0.5 ps was used. Coordinates were saved every 100 frames (i.e. 500 ps).

The effective diffusion coefficients used in Eq.(5) were obtained from standard many-solute SDA simulations following the protocol of Mereghetti and Wade (19). For each system, 100 BD simulations of 6,000,000 ps were performed, and the diffusion coefficients were extracted by fitting the mean-squared displacement of the molecule of interest over time, as described in (20). These simulations employed the same precomputed interaction grids and parameters detailed above. The resulting diffusion coefficients are reported in Fig. S3.

Reaction criteria. In order to calculate the probability β of 'reacting' before 'escaping' (Eq.(5)), it is necessary to define reaction criteria. To establish these, all polar contacts between the receptor protein and the ligand in the holo-structure were identified, i.e. hydrogen bond acceptor-donor pairs. Selected non-polar contacts were also included as reaction criteria, namely: halogen-aromatic, aromatic-aromatic $\pi - \pi$, methyl-aromatic, non-aromatic ring $-\pi$, between proteins and ligands in the holo-structures. The coordinates of these contact atoms were then extracted and added to a reaction criteria list when the distance between them was less than 3.5 Å for hydrogen-bonded pairs and less than 4.5 Å for the other interactions. Since BD simulations were performed using the apo-form of the receptor protein with a rigid-body representation, a global sequence alignment of the holo- and apo-structures employing the Needleman-Wunsch algorithm was required. This alignment enabled the identification of the holo-protein atoms involved in the contacts and their replacement with the corresponding atoms in the apo-structure in the reaction criteria list.

Based on this reaction list, an encounter complex was defined as a configuration in which all the independent native contacts of the receptor-ligand complex are simultaneously formed within a specified distance threshold. Native contacts were considered independent if the corresponding interacting atoms on the receptor protein were separated by more than 2 Å. The distance threshold for satisfying the independent contacts of the reaction criteria during the BD simulations was selected as the minimum distance at which at least 20 trajectories were observed to result in encounter complex formation in the infinitely dilute solution condition. This definition resulted in reaction criteria of three independent contacts at 6 Åfor the trypsin-benzamidine system and seven independent contacts at 13.5 Åfor the haspin-chlorotubercidin system.

Trajectory analysis. Trajectories were analyzed using MSM to investigate ligand binding pathways. For MSM construction, only parts of the subset of trajectories in which the reaction criteria for encounter complex formation were satisfied were considered. These trajectory segments were defined as the segments beginning from the first frame in which at least one ligand atom is within 6.5 Å of any receptor protein atom, and ending when the required independent contacts in the reaction list are simultaneously satisfied, indicating encounter complex formation. This approach enables the identification of transient or secondary binding sites on the receptor protein surface that may serve as intermediate states on the pathways to the primary

binding pocket.

MSMs were constructed using the Python library deeptime(21). The number of clusters and the lag time were determined based on analysis of the Within-Cluster Sum of Squares (WCSS) and the implied timescales of the transition matrix eigenvalues as a function of lag time (see Fig.S4 in Supporting Information). This procedure resulted in the selection of 6 clusters and a lag time of 4 saved frames. In the BD simulations, the integration timestep was 0.5 ps and coordinates were saved every 100 timesteps (corresponding to 50 ps between saved frames); therefore, using a lag time of 4 steps for MSM construction means that transitions are counted between frames separated by 200 ps.

The MSM results were visualized by plotting the spatial distribution of the ligand position clusters on the protein surface, together with representative MSM graphs. In these graphs, node size reflects the stationary probability of the ligand being in each cluster (i.e., the total number of associated microstates), and arrow thickness represents the transition probability between clusters. As simulations at different crowder densities yield independent sets of encounter trajectories, the resulting MSMs are not directly comparable in terms of node size or transition probability scale; the graphical elements are only meaningful within each individual model. In addition, since the simulations used for this analysis end upon binding, the MSMs capture only the encounter phase and do not represent full binding pathways.

To investigate the arrangement of the crowders around the ligand binding pocket, CAVER(22, 23) (version 3.0.3)— a tool for detecting tunnels in protein structures— was employed. This analysis enabled the assessment of whether crowders influence the receptor-ligand binding process through adopting specific spatial arrangements near the entrance of the binding pocket. As the PyMOL(24) plugin for CAVER requires a structure as input for tunnel detection, the last ten saved frames from encounter trajectories of the highest-density simulations were selected. Since the BD timestep is 0.5 ps and coordinates were saved every 100 steps, these correspond to the last 500 ps of the trajectories, with frames spaced by 50 ps. From each frame, only the receptor protein (either trypsin or haspin) and the crowders located within a 10 Å radius of the ligand were retained. Across all sampled frames, the maximum number of crowders present within this 10 Å threshold was consistently two (see Fig. S14 for the underlying distance distributions). We specifically focused the CAVER analysis on these two-crowder configurations as they provide the most topologically relevant landscapes for the characterization of localized channeling effects. For tunnel detection, the starting point was defined as the midpoint between specific receptor protein residues located at the entrance to the binding pocket. In trypsin, this point was set between the OG atom of SER195 and the O atom of GLY216. For haspin, it was defined between the O atom of LYS128 and the OE2 atom of GLU167. The probe to represent the ligand was assigned a minimum radius of 3 Å for benzamidine and 4 Å for chlorotubercidin. All other CAVER parameters were kept at their default values.

Supporting Figures

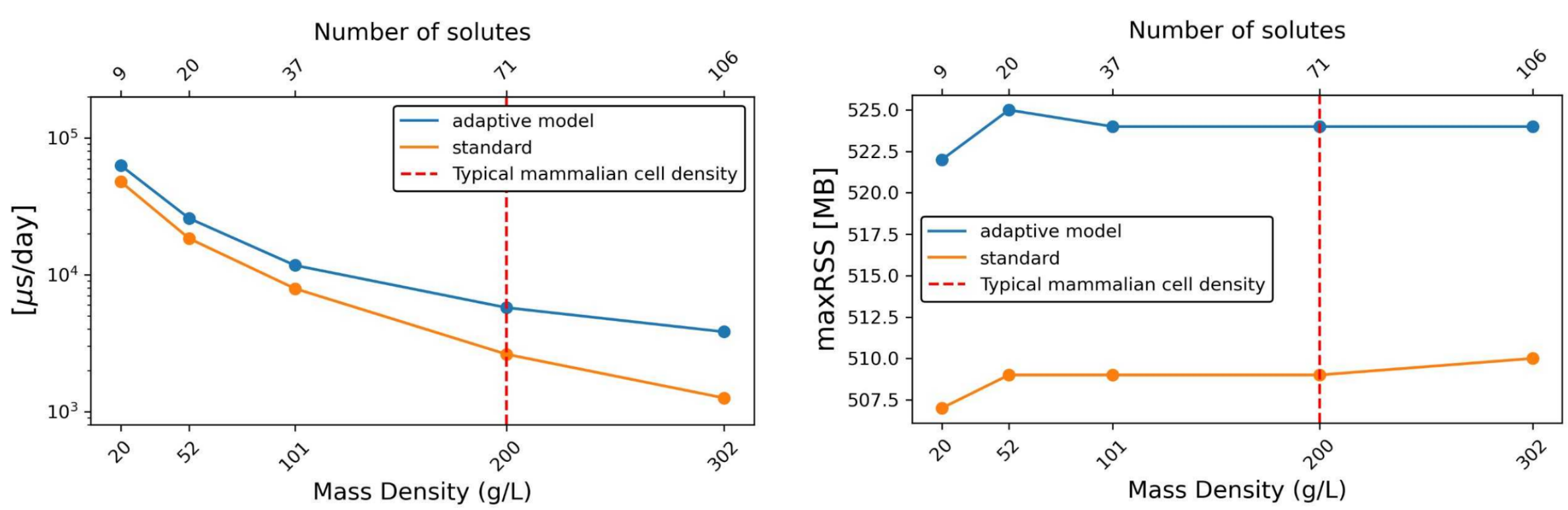

Fig. S1. Comparison of the computational cost in terms of efficiency (left) and memory use (right) between Brownian dynamics simulations performed with and without the adaptive model. The simulations are for the association of trypsin and benzamidine in the presence of charged lysozyme crowders in a periodic simulation box with a dimension of 200 Å. Computational efficiency is reported in nanoseconds per day, while memory use is reported as maxRSS (maximum amount of memory occupied by a given job in Mbytes). The simulations with the adaptive model at a crowder density of 200 g/L are up to three times faster than the standard model without the adaptive model, with a small increase in memory usage of 2.95%. The reason for the small increase in maxRSS is due to the storage of 1-dimensional interaction grids for crowder interactions at the coarse-grained resolution. The BD simulations were run in parallel with OpenMP using 8 CPUs on one compute node (Intel (R) Xeon(R) Gold 6230 2.10GHz CPU; x86_64).

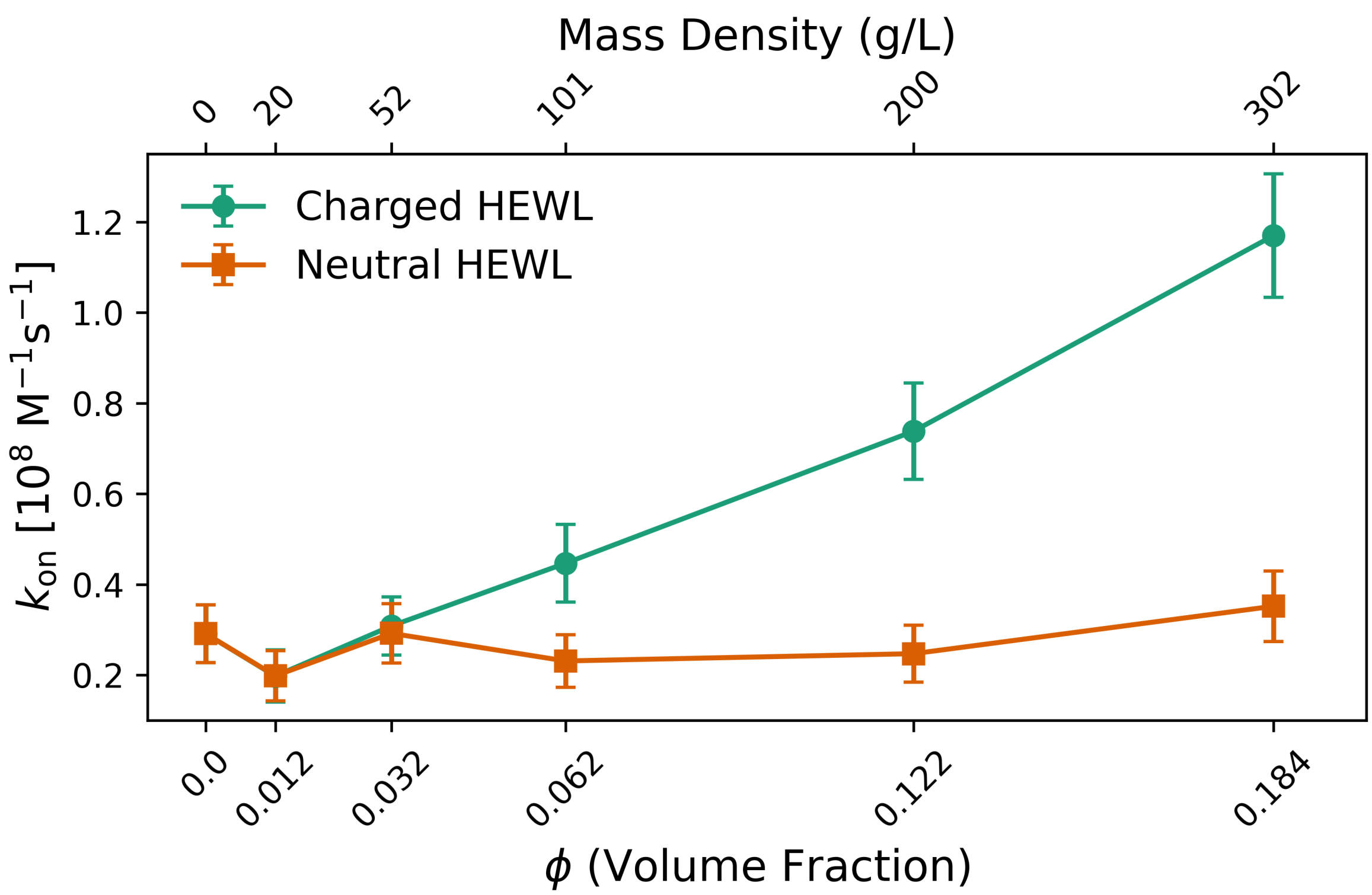

Fig. S2. Comparison of computed partially crowded bimolecular association rate constants for simulations with forces computed with (labeled "adaptive model") and without (labeled "standard") the adaptive model. The association rate constants computed with the adaptive model all fall within the error bars of the benchmark values computed without use of the adaptive model. Thus, the efficiency gains from the two-resolution approach employing the adaptive model do not come at the expense of precision in calculating the association rate constant.

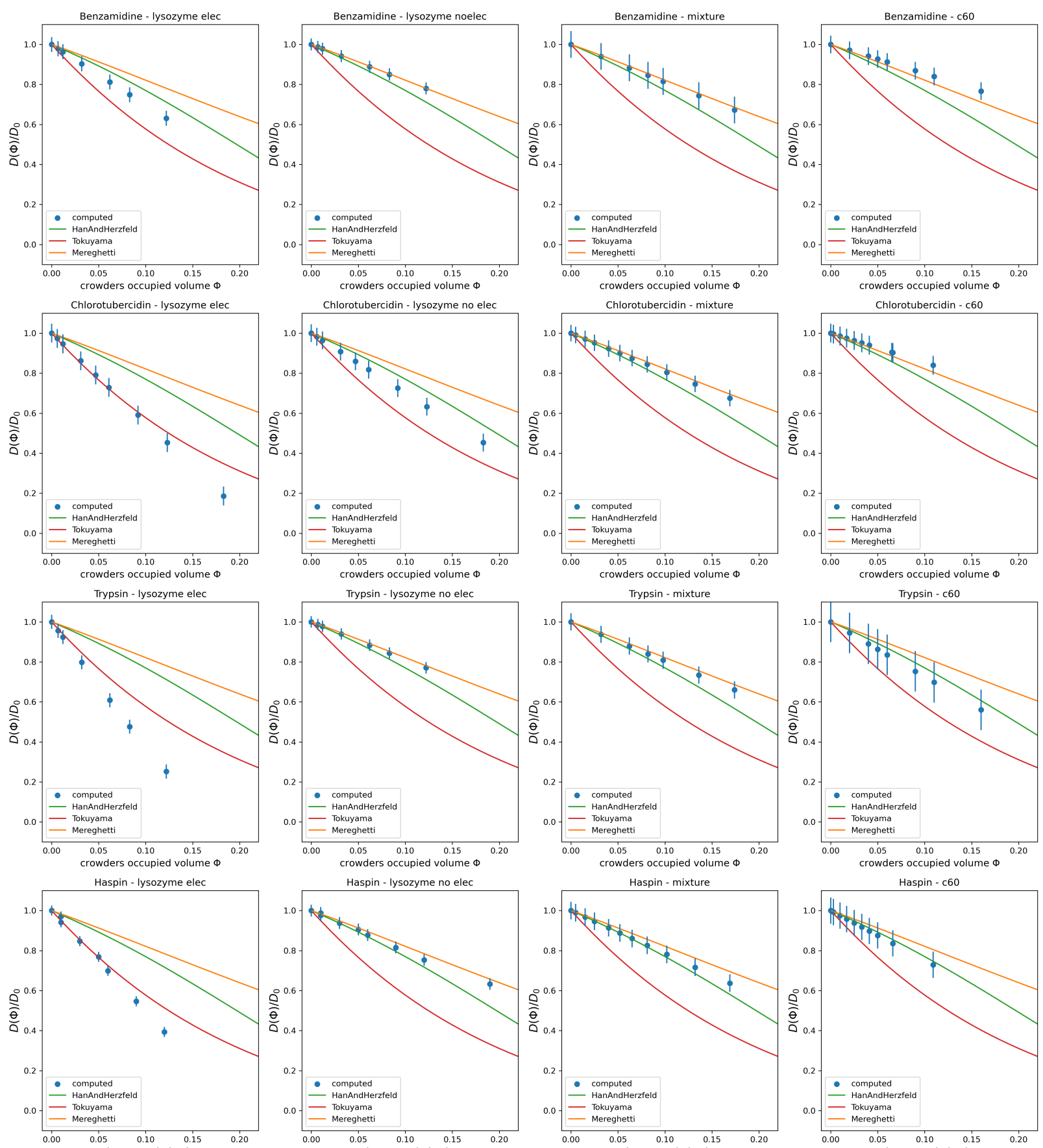

Fig. S3. Computed translational diffusion coefficients of the non-crowders as a function of crowder concentration. Values from BD simulations (blue dots with standard deviations) were used to compute association rate constants according to Eq.(5) of the main text and are shown alongside three empirical models for hard-sphere suspensions (20). The three continuum lines represent different models which are described in detail in (20). In brief, the "Mereghetti" model corresponds to local volume fraction-dependent short-time translational diffusion including hydrodynamic interactions implemented in (20); the Han & Herzfeld model (25) is based on scaled particle theory (SPT) and describes long-time diffusion due to steric crowding only; and the Tokuyama model (1) includes both direct and hydrodynamic interactions. Simulation results with all interactions (crowders charged) follow the Tokuyama model, as expected, because it accounts for both direct (steric and electrostatic) and hydrodynamic interactions. When electrostatic interactions are removed, the results shift closer to the Han & Herzfeld SPT prediction, reflecting the reduced contribution of direct interactions. This behavior is consistent with the findings of Mereghetti et al. (19).

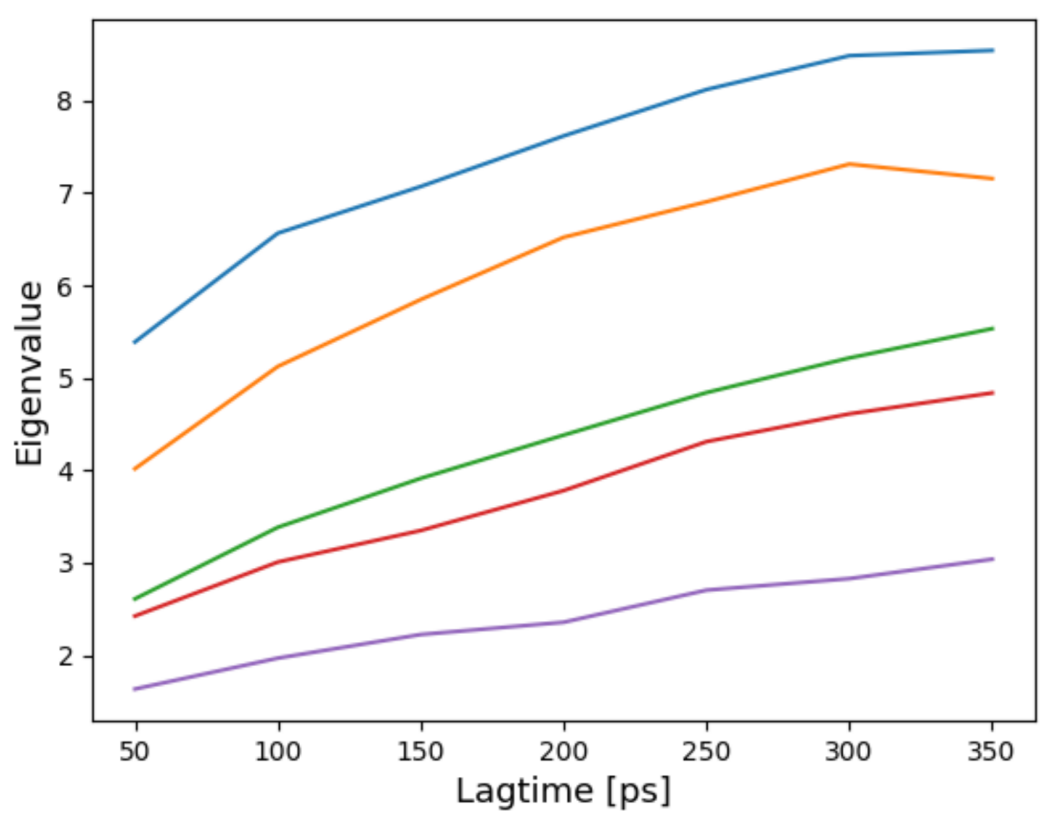

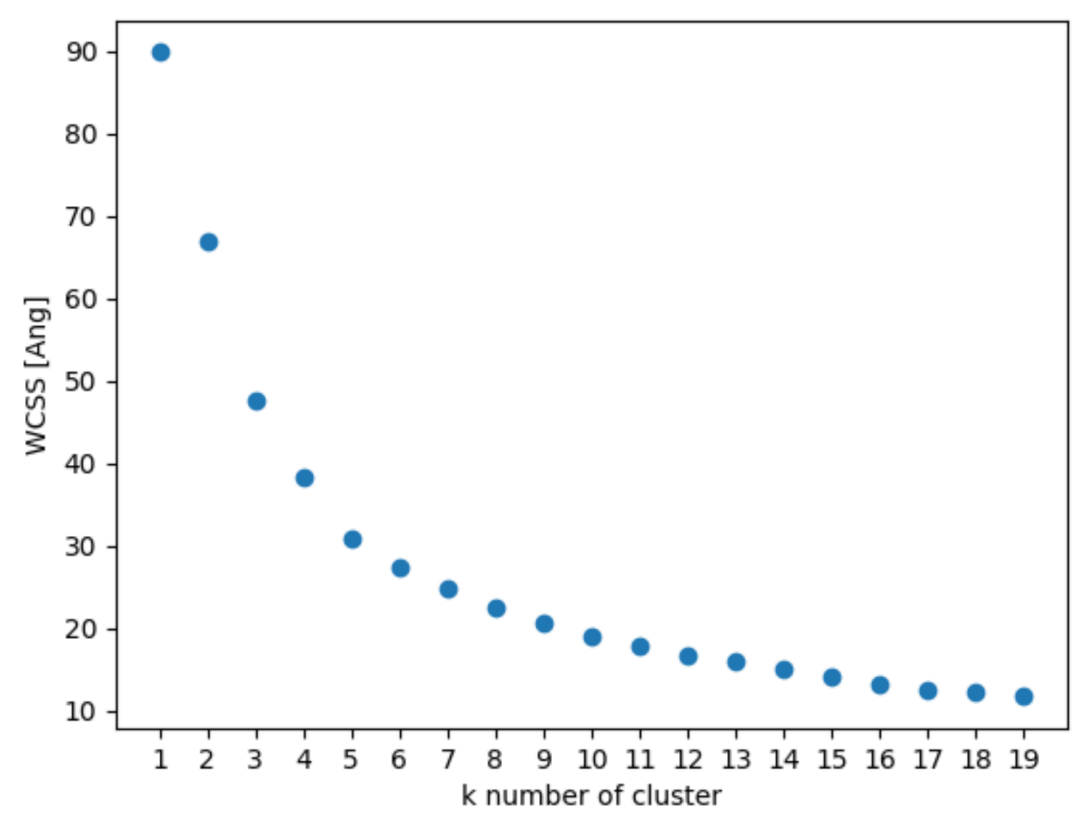

Fig. S4. Example of model selection for construction of a Markov State Model (MSM) for receptor-ligand association. Results are shown for trypsin-benzamidine association in the presence of neutralized HEWL at a crowder volume fraction of $\phi = 0.184$. (Left): Eigenvalue spectra as a function of lag time for the dynamic modes. Each colored line corresponds to one of the five eigenvectors associated with the highest eigenvalues. Eigenvalues are dimensionless while lag times are reported in ps. Unlike for a standard equilibrium MSM, the eigenvalues do not exhibit a clear plateau with increasing lag time. This behavior is expected given the conditional nature of the MSM model: trajectories are required to result in encounter and they are truncated upon reaching a defined encounter or bound state, introducing a probability sink and breaking detailed balance. Consequently, the system does not sample the full equilibrium distribution, and implied timescales cannot fully converge. (Right): Plot of the within-cluster sum of squares (WCSS) as a function of the number of clusters (k) which is used to determine the number of macrostates in the MSM. An appropriate number of clusters can be chosen by locating the point where increasing k yields only a marginal decrease in WCSS, indicating diminishing returns in clustering quality.

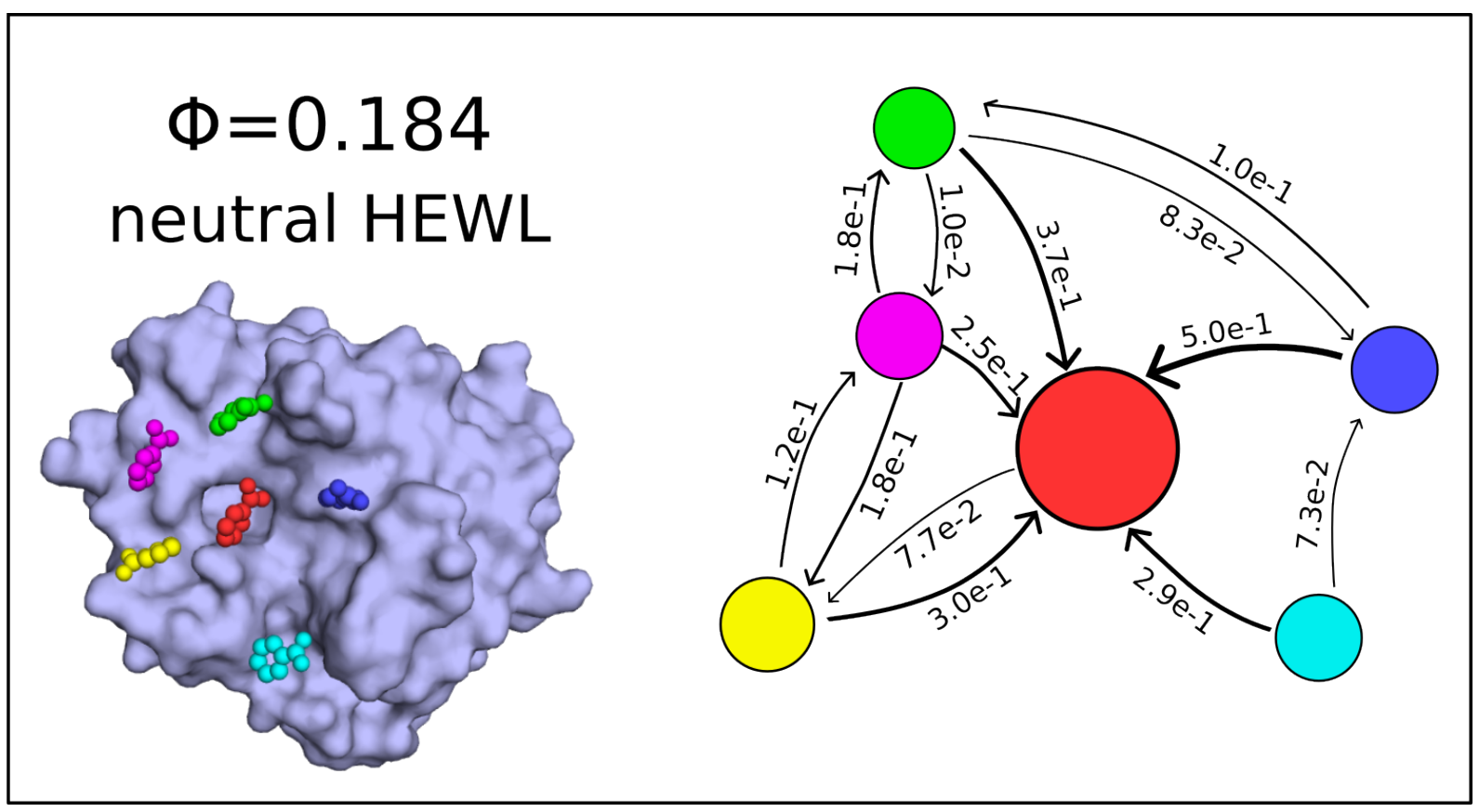

Fig. S5. MSM model computed for encounter trajectories of benzamidine binding to trypsin in the presence of neutralized HEWL crowders. Benzamidine takes a rather direct route to the bound state (red cluster) with other clusters being small and rather close to the bound state.

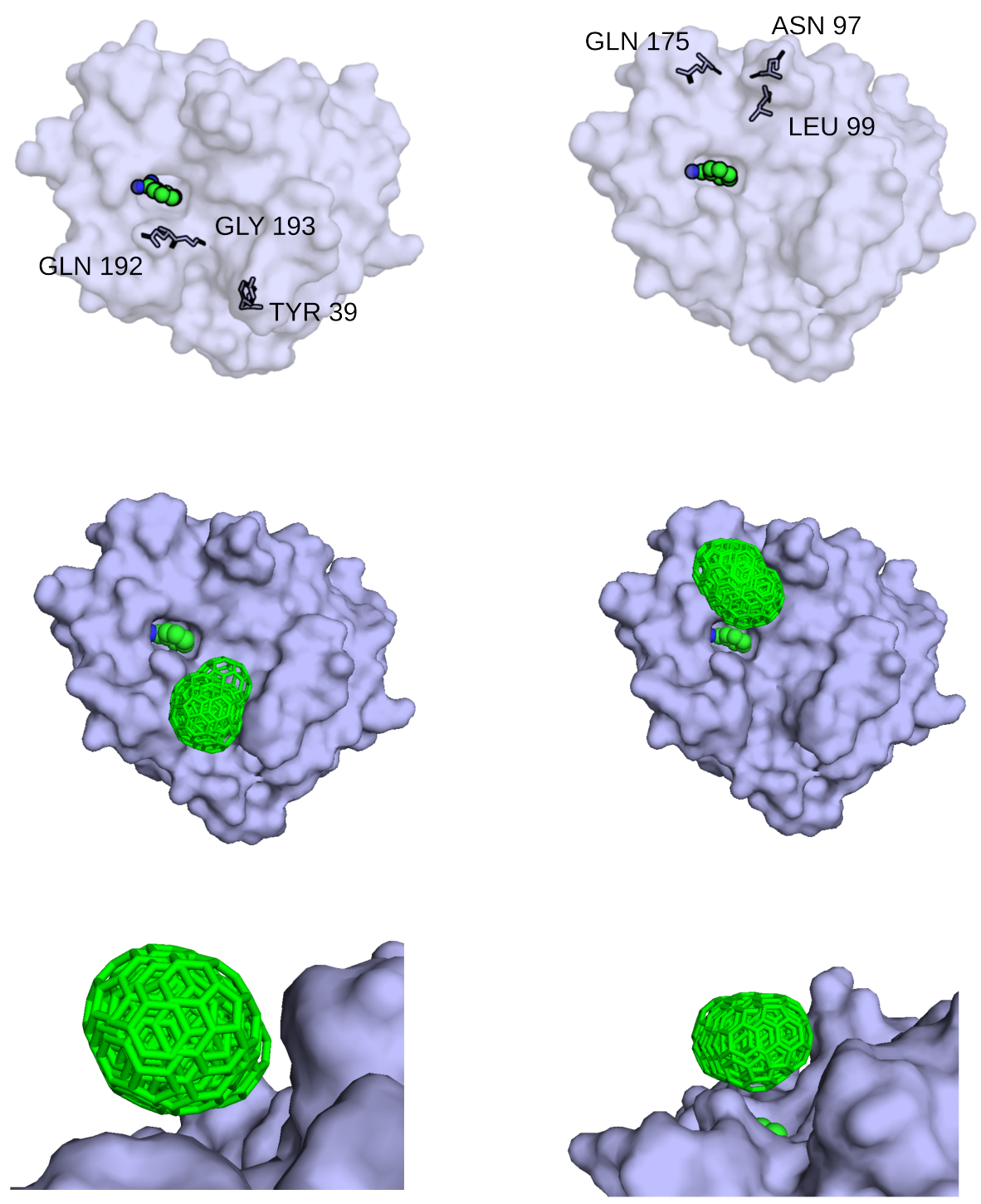

Fig. S6. Fullerene C60 crowders nestle in two concave regions on the surface of trypsin. The upper images show the residues lining these regions and their location with respect to the binding site, which is shown with benzamidine (van der Waals sphere representation) bound. The middle images show the superposition of C60 structures from a hundred snapshots from the simulations performed at $\phi = 0.6$ (the crowding density resulting in the lowest computed association rate constant), illustrating how well localized the fullerene is on the trypsin surface. The lower images show corresponding close-ups viewed from the side (rotated about 90 degrees into the page).

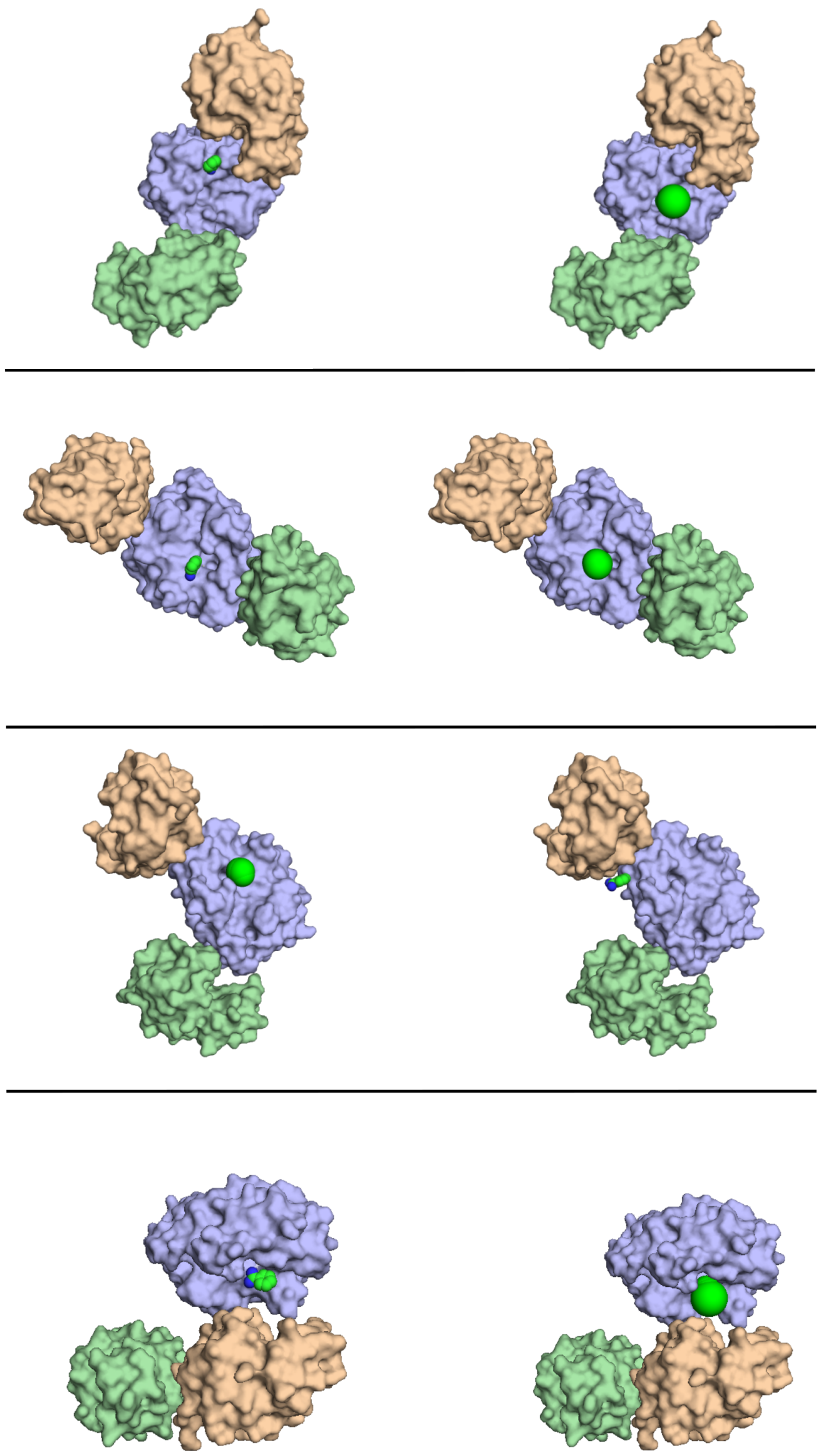

Fig. S7. CAVER analysis of tunnels in eight late stage snapshots of encounter trajectories for benzamidine binding to trypsin (lilac) in the presence of charged HEWL crowders (green and orange). The CAVER route to the trypsin active site for a spherical probe the size of benzamidine is shown in green. The absence of specific interactions between trypsin and the HEWL crowders means that there is no channeling effect on benzamidine.

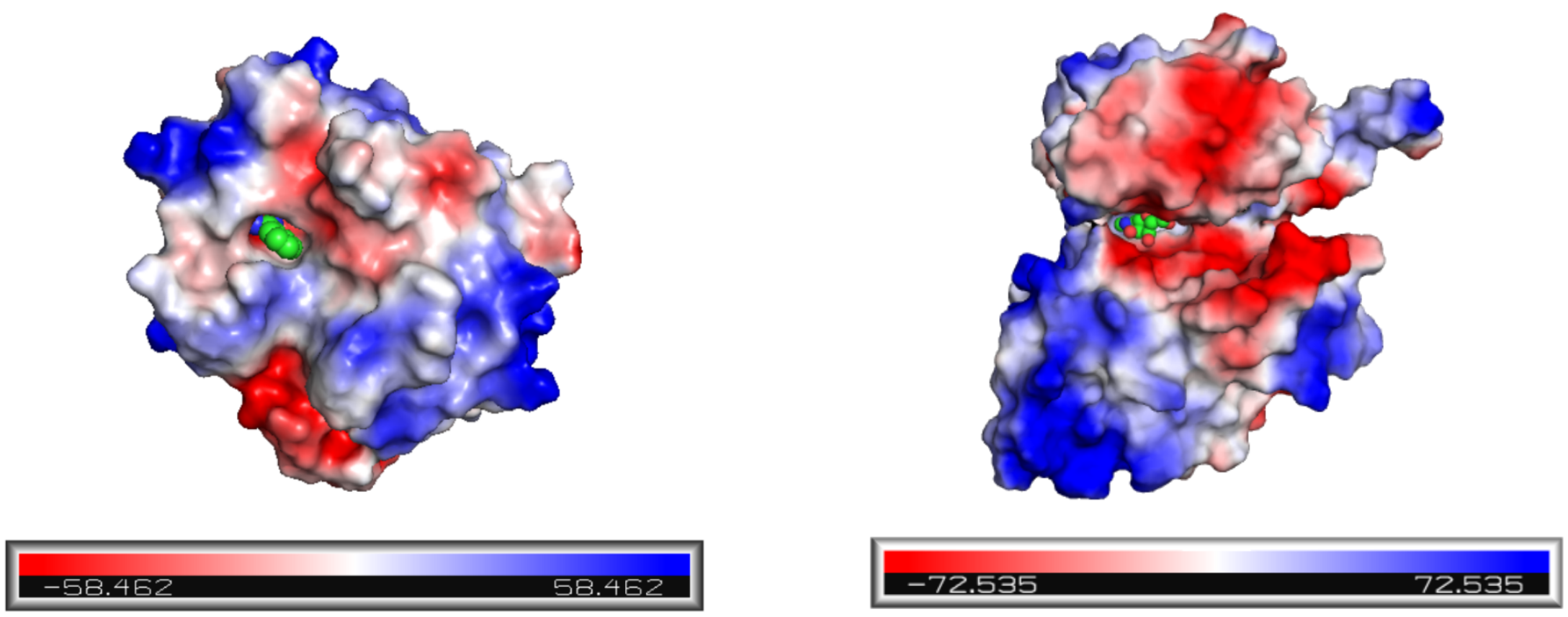

Fig. S8. Electrostatic potential of trypsin (left) and haspin (right). The electrostatic potentials (kT/e units, pH 7 and zero ionic strength) are mapped onto their molecular surfaces, with the binding pockets shown containing benzamidine and chlorotubercidin, respectively. In contrast to haspin, the absence of large positively or negatively charged regions near the trypsin binding pocket does not facilitate specific interactions with the positively charged HEWL crowders. This may explain the absence of a channeling effect of the HEWL crowders on the trypsin-benzamidine association.

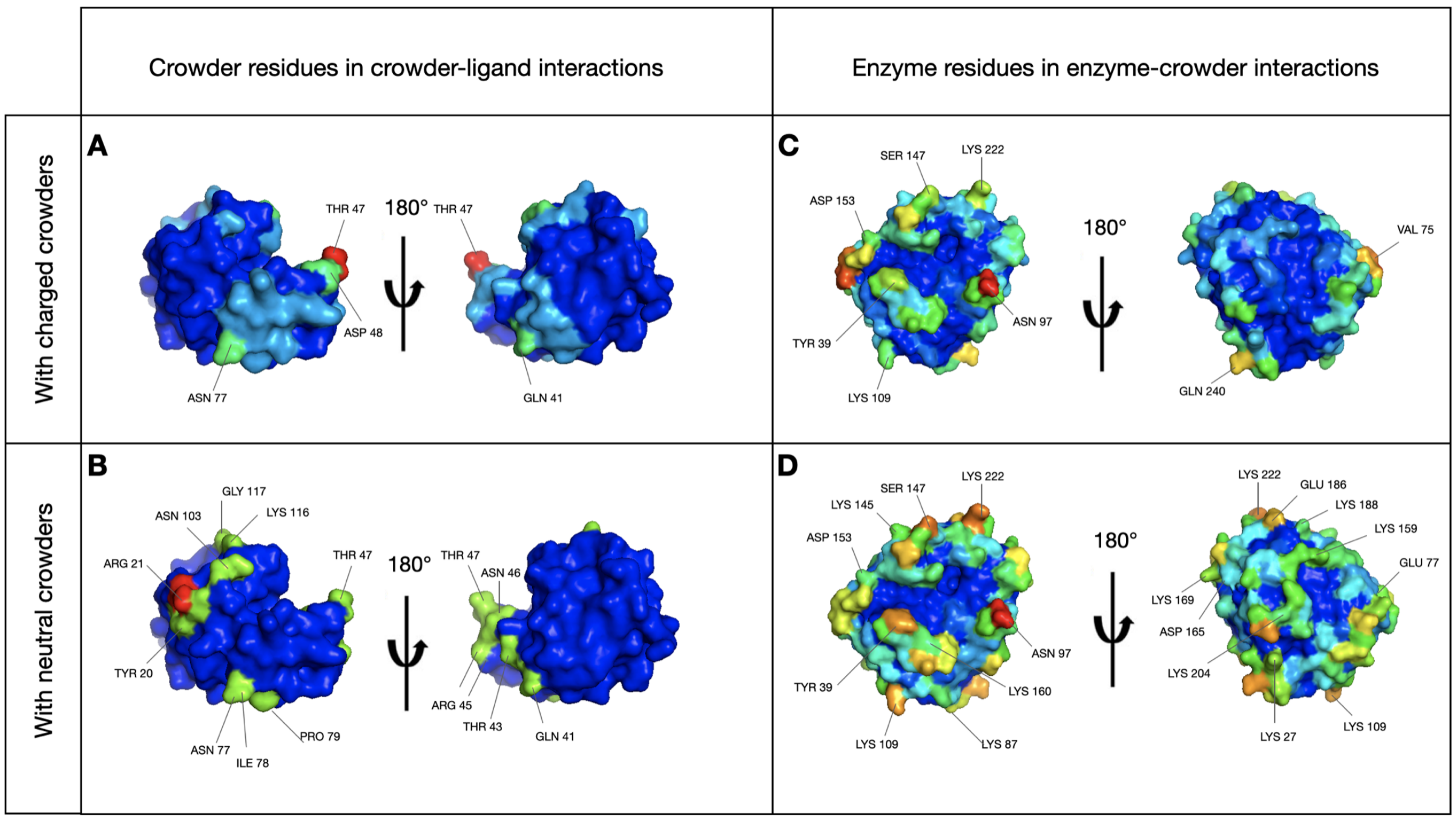

Fig. S9. Residue-level contact frequency mapping for crowder–ligand and enzyme–crowder interactions in the trypsin–benzamidine system. The contact frequencies obtained from the Brownian dynamics trajectories are mapped onto the molecular surfaces using a color gradient from blue (no/low interaction frequency) to red (high interaction frequency). (A, B) Spatial distribution of crowder (HEWL) residues involved in direct interactions with benzamidine in the presence of (A) charged and (B) neutral crowders. The charged ensemble features a significantly restricted interaction footprint, prominently driven by electrostatic repulsion between the positively charged ligand and basic crowder residues such as ARG 21. (C, D) Spatial distribution of trypsin residues involved in direct contact with (C) charged and (D) neutral HEWL crowders. The position of the catalytic binding pocket is denoted by a white circle labeled "b". While the contact frequencies directly surrounding the active site entrance remain comparable, a stark contrast is observed on the opposite face of the enzyme (shown by a 180° rotation). In the presence of charged HEWL crowders (C), a cluster of basic surface residues (including LYS 27, LYS 109, LYS 169, LYS 188, LYS 204, and LYS 222) exerts strong electrostatic repulsion on the charged HEWL molecules, reducing back-face coordination and asymmetrically forcing the crowders toward the front-face with the binding site to establish an effective electrostatic cage.

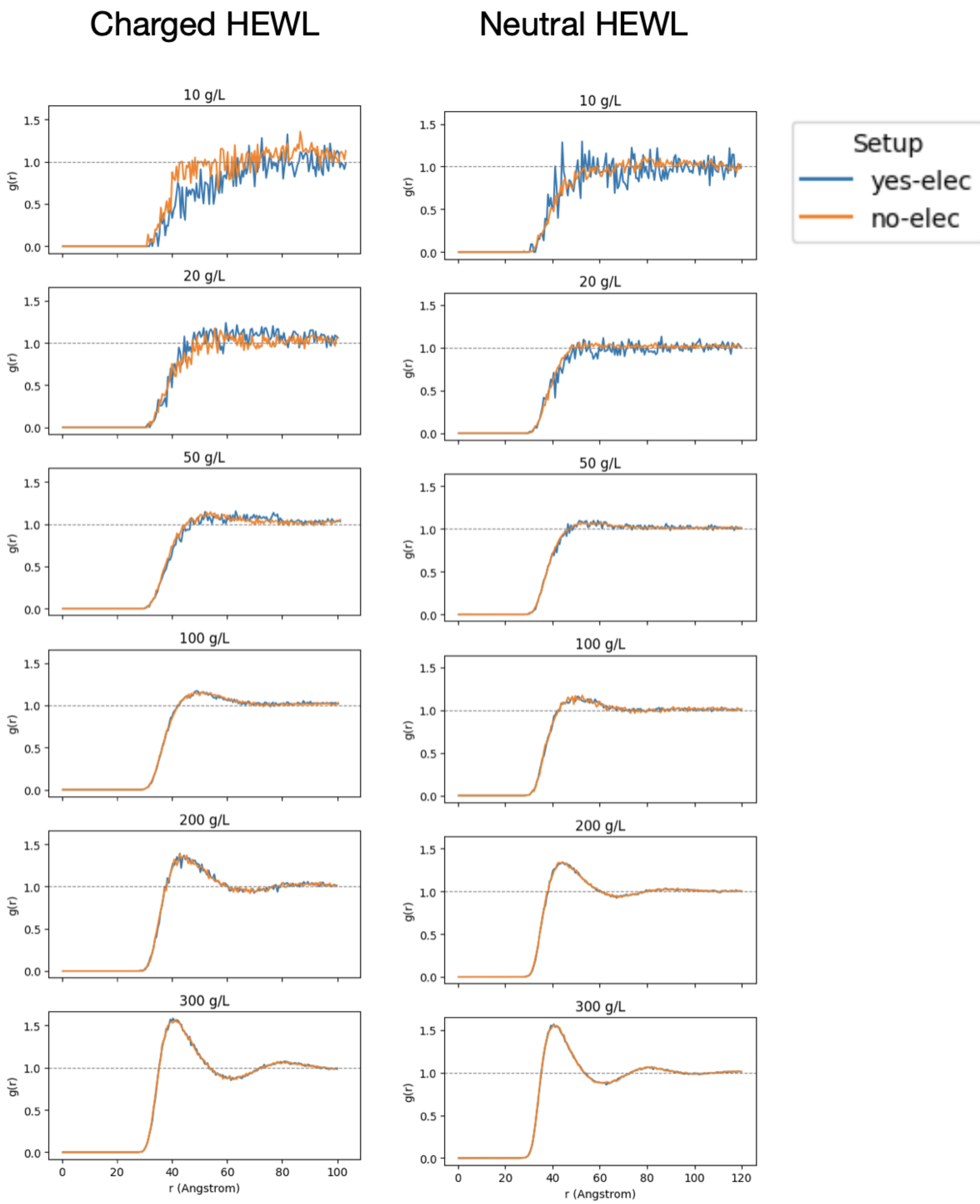

Fig. S10. Crowder-crowder radial distribution functions for charged and neutral lysozymes at different densities. Left, trypsin-benzamidine system. Right, haspin-chlorotubercidin system. Radial distribution functions were computed from a single trajectory file for each crowder density.

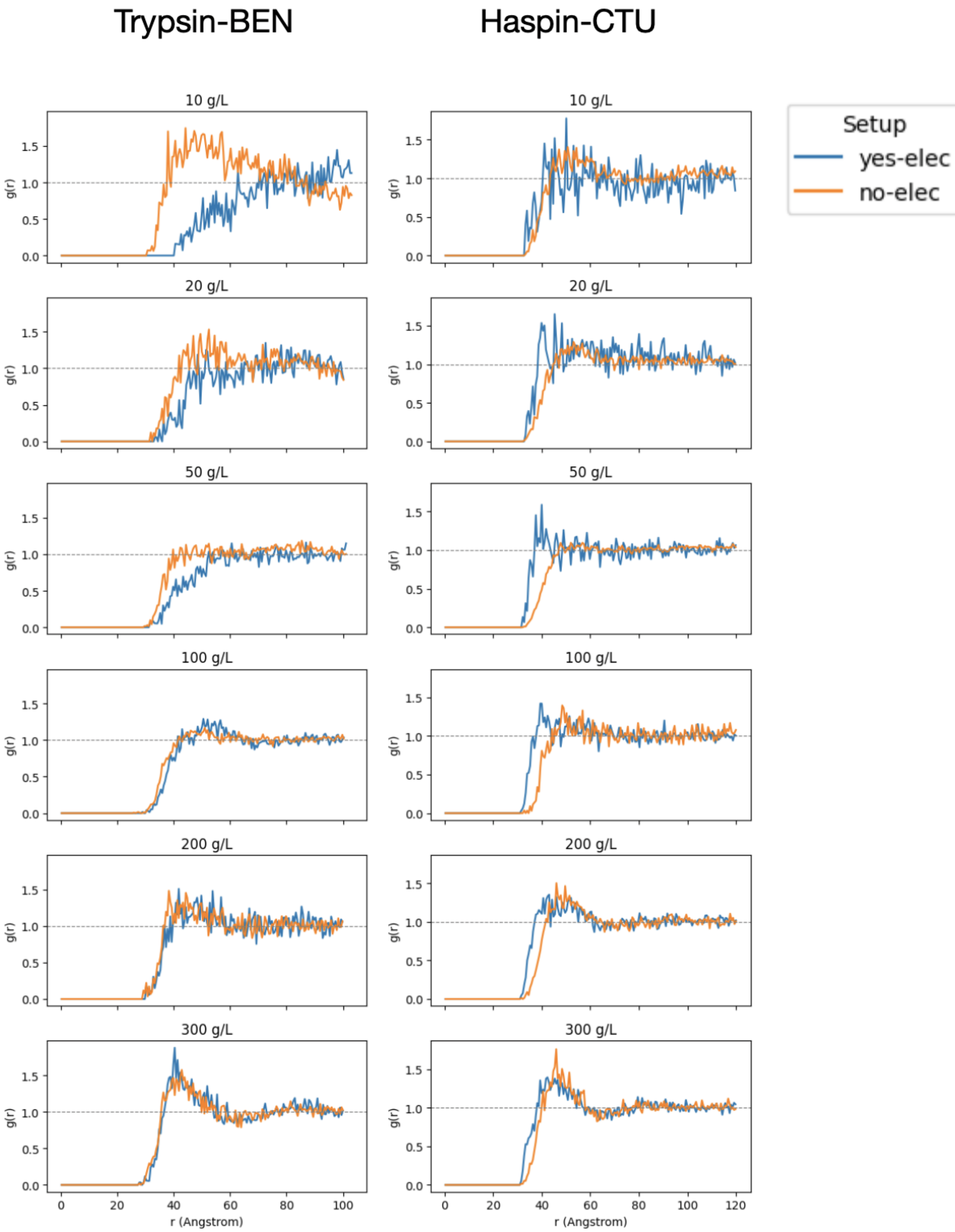

Fig. S11. Crowder-enzyme radial distribution functions for charged and neutral lysozymes at different crowder densities . Left, trypsin:benzamidine system. Right, haspin: chlorotubercidin system. Radial distribution functions were computed from a single trajectory file for each crowder density.

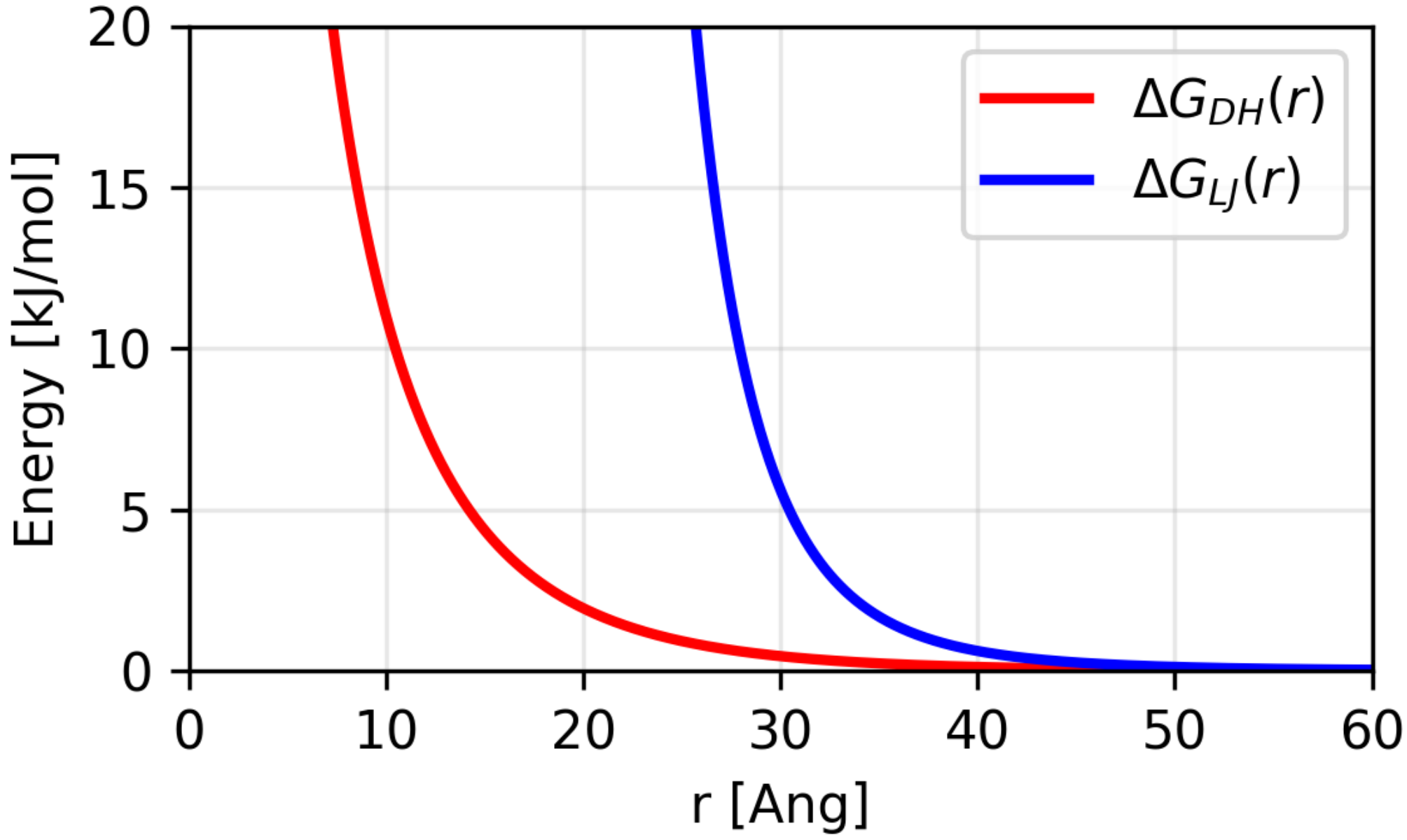

Fig. S12. Energetic balance between electrostatic repulsion and short-range Lennard-Jones repulsion. The distance dependence of the Debye–Hückel electrostatic energy, $\Delta G_{DH}(r)$ (red), and the adaptive-model Lennard-Jones repulsive energy, $\Delta G_{LJ}(r)$ (blue) between two charged crowders is plotted. The parameters of the potentials corresponding to the HEWL crowders used in this study (hydrodynamic radii $a_1 = a_2 = 15$ Å, net charge $z_1 = z_2 = +8e$) at the ionic strength of 100 mM (Debye screening parameter $k = 0.104\text{Å}^{-1}$). Under these conditions, the Lennard-Jones repulsive term dominates over the screened electrostatic repulsion at all separations where the two terms are non-negligible, so ΔG_{LJ} crosses below 20 kJ/mol only once $\Delta G_{DH}(r)$ has already decayed to near zero — explaining why the charged and neutral HEWL crowder–crowder RDFs are indistinguishable (Fig. S10).

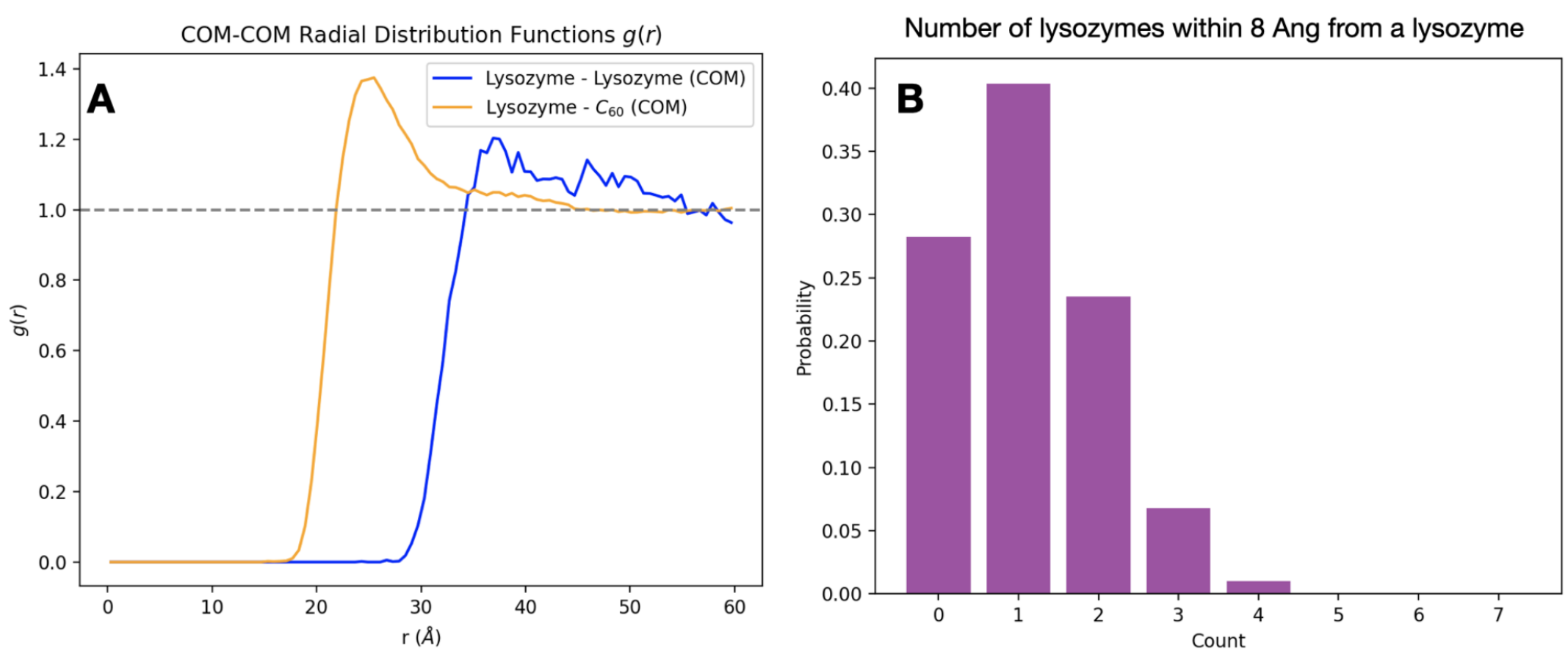

Fig. S13. Phase behavior, structural arrangement, and mixing homogeneity in the multi-component mixed crowder system. (A) Center-of-mass (COM) radial distribution functions, g(r), as a function of inter-particle distance for homotypic lysozyme–lysozyme (blue line) and heterotypic lysozyme–C60 fullerene (orange line) pairs. The distinct, sharp heterotypic peak observed at $r \sim 25$ Å highlights the smooth structural organization of fullerenes into a well-defined solvation shell surrounding the lysozyme molecules, rather than local phase segregation. At long range, both distribution functions asymptotically approach unity ($g(r) = 1.0$), confirming a completely homogeneous, ideal-gas-like mixing state without macro- or micro-phase separation. (B) Probability distribution of the local coordination number, representing the absolute number of neighboring lysozyme molecules found within a close-contact radius of 8 Å from a reference lysozyme. Over 65% of the sampled configurations show a coordination number of one or zero, demonstrating the absence of self-aggregation or homotypic protein clumping within the mixed crowded matrix. Radial distribution functions are computed from a single trajectory file for the trypsin-benzamidine system with a mixture crowders at $\phi = 0.174$ (same system as reported in Fig.1).

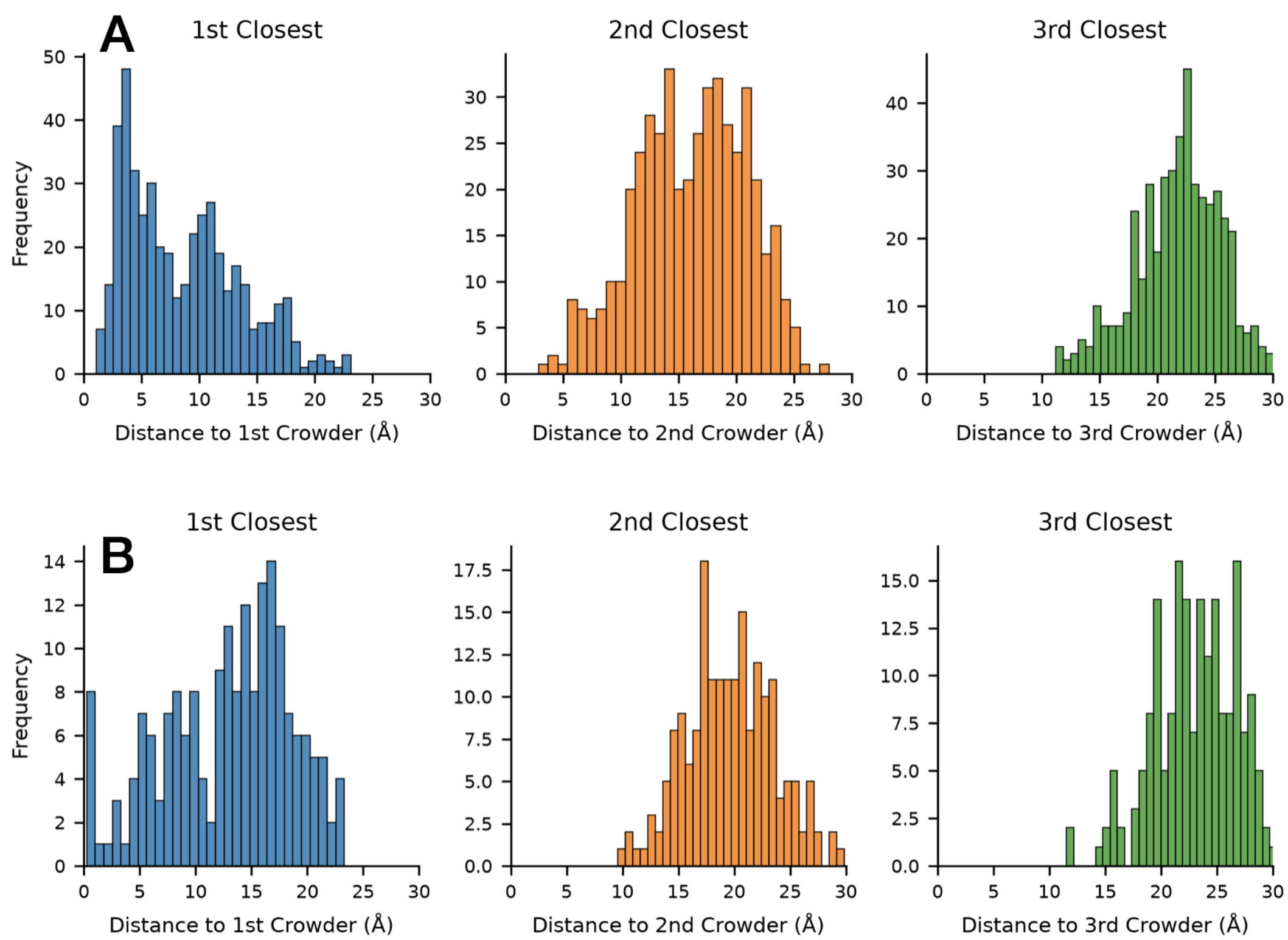

Fig. S14. Distance distribution profiles of the nearest crowders during ligand binding encounters in the presence of charged (A) and neutral (B) HEWL crowders. Histograms display the frequency of the minimum distance (in Å) between the ligand and the first (left, blue), second (middle, orange), and third (right, green) closest lysozyme crowders across the analyzed encounter frames of the haspin–chlorotubercidin system.

Supporting Tables

Table S1. Crystal structures of the proteins used for the simulations

Unbound protein	Protein-ligand complex
Trypsin<br>pdb: 5MNF(26)	Trypsin – benzamidine<br>pdb: 4I8H(27)
Haspin<br>pdb: 2WB8(28)	Haspin – 5-chlorotuberdicin<br>pdb: 6G36(14)
HEWL<br>pdb: 1HEL(29)	

Table S2. Extents of the interaction grids used for computing intermolecular forces in the BD simulations. For all grids, the grid spacing is 1 Å, except for the lj_rep grid, for which the grid spacing is 0.5 Å to avoid excessive overlap between molecules due to discretization.

Molecule	elec_pot [Å]	elec_des [Å]	hd_des [Å]	lj_rep [Å]
**Benzamidine**	65	110	110	20
**Haspin**	129	110	110	110
**Chlorotuberdicin**	65	110	110	20
**HEWL**	129	80	60	80
**C60**	-	-	15	15

References

1. M Tokuyama, T Moriki, Y Kimura, Self-diffusion of biomolecules in solution. *Phys. Rev. E* **83**, 051402 (2011).
2. B Cichocki, M Ekiel-Jeżewska, E Wajnryb, Lubrication corrections for three-particle contribution to short-time self-diffusion coefficients in colloidal dispersions. *J. Chem. Phys.* **111**, 3265–3273 (1999).
3. HM Berman, et al., The protein data bank. *Nucleic acids res.* **28**, 235–242 (2000) (URL: www.rcsb.org).
4. G Madhavi Sastry, M Adzhigirey, T Day, R Annabhimoju, W Sherman, Protein and ligand preparation: parameters, protocols, and influence on virtual screening enrichments. *J. Comput. Aided Mol. Des.* **27**, 221–234 (2013).
5. TJ Dolinsky, JE Nielsen, JA McCammon, NA Baker, Pdb2pqr: an automated pipeline for the setup of poisson–boltzmann electrostatics calculations. *Nucleic Acids Res.* **32**, W665–W667 (2004).
6. JW Ponder, DA Case, Force fields for protein simulations. *Adv. Protein Chem.* **66**, 27–85 (2003).
7. C Lu, et al., Opls4: Improving force field accuracy on challenging regimes of chemical space. *J. Chem. Theory Comput.* **17**, 4291–4300 (2021).
8. GK Ganotra, "Computational studies of drug-binding kinetics," PhD thesis, Heidelberg University (2020).
9. A Muñiz-Chicharro, GK Ganotra, RC Wade, A multiscale simulation approach to compute protein–ligand association rate constants by combining brownian dynamics and molecular dynamics. *J. Chem. Inf. Model.* **65**, 11215–11231 (2025).
10. GM Barca, et al., Recent developments in the general atomic and molecular electronic structure system. *J. Chem. Phys.* **152** (2020).
11. BR Dandekar, BB Majumdar, J Mondal, Nonmonotonic modulation of the protein–ligand recognition event by inert crowders. *J. Phys. Chem. B* **127**, 7449–7461 (2023).
12. E Jurrus, et al., Improvements to the apbs biomolecular solvation software suite. *Protein Sci.* **27**, 112–128 (2018).
13. F Guillain, D Thusius, Use of proflavine as an indicator in temperature-jump studies of the binding of a competitive inhibitor to trypsin. *J. Am. Chem. Soc.* **92**, 5534–5536 (1970).
14. C Heroven, et al., Halogen–aromatic π interactions modulate inhibitor residence times. *Angew. Chem. Int. Ed. Engl.* **57**, 7220–7224 (2018).
15. RR Gabdoulline, RC Wade, Effective charges for macromolecules in solvent. *J. Phys. Chem.* **100**, 3868–3878 (1996).
16. RR Gabdoulline, RC Wade, On the contributions of diffusion and thermal activation to electron transfer between phormidium laminosum plastocyanin and cytochrome f: Brownian dynamics simulations with explicit modeling of nonpolar desolvation interactions and electron transfer events. *J. Am. Chem. Soc.* **131**, 9230–9238 (2009).
17. JG de la Torre, ML Huertas, B Carrasco, Calculation of hydrodynamic properties of globular proteins from their atomic-level structure. *Biophys. J.* **78**, 719–730 (2000).
18. D Dey, A Nunes-Alves, R Wade, G Schreiber, Diffusion of small molecule drugs is affected by surface interactions and crowder proteins. iscience 25: 105088. *iScience* (2022).
19. P Mereghetti, RR Gabdoulline, RC Wade, Brownian dynamics simulation of protein solutions: structural and dynamical properties. *Biophys. J.* **99**, 3782–3791 (2010).
20. P Mereghetti, RC Wade, Atomic detail brownian dynamics simulations of concentrated protein solutions with a mean field treatment of hydrodynamic interactions. *J. Phys. Chem. B* **116**, 8523–8533 (2012).

21. M Hoffmann, et al., Deeptime: a python library for machine learning dynamical models from time series data. *Mach. Learn. Sci. Technol.* (2021).
22. E Chovancova, et al., Caver 3.0: A tool for the analysis of transport pathways in dynamic protein structures. *PLoS Comput. Biol.* **8**, 1–12 (2012).
23. A Pavelka, et al., Caver: Algorithms for analyzing dynamics of tunnels in macromolecules. *IEEE/ACM Trans. Comput. Biol. Bioinform.* **13**, 505–517 (2015).
24. Schrödinger, LLC, W DeLano, PyMOL (2020) Version 2.6.0.
25. J Han, J Herzfeld, Macromolecular diffusion in crowded solutions. *Biophys. J.* **65**, 1155–1161 (1993).
26. J Schiebel, et al., Intriguing role of water in protein-ligand binding studied by neutron crystallography on trypsin complexes. *Nat. Commun.* **9**, 3559 (2018).
27. D Liebschner, M Dauter, A Brzuszkiewicz, Z Dauter, On the reproducibility of protein crystal structures: five atomic resolution structures of trypsin. *Acta Crystallogr. Sect. D: Biol. Crystallogr.* **69**, 1447–1462 (2013).
28. F Villa, et al., Crystal structure of the catalytic domain of haspin, an atypical kinase implicated in chromatin organization. *Proc. Natl. Acad. Sci. U.S.A* **106**, 20204–20209 (2009).
29. KP Wilson, B Malcolm, B Matthews, Structural and thermodynamic analysis of compensating mutations within the core of chicken egg white lysozyme. *J. Biol. Chem.* **267**, 10842–10849 (1992).
